# Identifying the p65-Dependent Effect of Sulforaphene on Esophageal Squamous Cell Carcinoma Progression via Bioinformatics Analysis

**DOI:** 10.3390/ijms22010060

**Published:** 2020-12-23

**Authors:** Sichong Han, Zhe Wang, Jining Liu, Qipeng Yuan

**Affiliations:** State Key Laboratory of Chemical Resource Engineering, College of Life Science and Technology, Beijing University of Chemical Technology, Beijing 100029, China; hansichong@126.com (S.H.); zhewangbuct@163.com (Z.W.); majiebeijinghos@163.com (J.L.)

**Keywords:** sulforaphene, esophageal squamous cell carcinoma, NFκB, *TNFAIP3*, *PLAU*

## Abstract

Understanding the mechanism by which sulforaphene (SFE) affects esophageal squamous cell carcinoma (ESCC) contributes to the application of this isothiocyanate as a chemotherapeutic agent. Thus, we attempted to investigate SFE regulation of ESCC characteristics more deeply. We performed gene set enrichment analysis (GSEA) on microarray data of SFE-treated ESCC cells and found that differentially expressed genes are enriched in TNFα_Signaling_via_the_NFκB_Pathway. Coupled with the expression profile data from the GSE20347 and GSE75241 datasets, we narrowed the set to 8 genes, 4 of which (C-X-C motif chemokine ligand 10 (*CXCL10*), TNF alpha induced protein 3 (*TNFAIP3*), inhibin subunit beta A (*INHBA*), and plasminogen activator, urokinase (*PLAU*)) were verified as the targets of SFE. RNA-sequence (RNA-seq) data of 182 ESCC samples from The Cancer Genome Atlas (TCGA) were grouped into two phenotypes for GSEA according to the expression of *CXCL10*, *TNFAIP3*, *INHBA*, and *PLAU*. The enrichment results proved that they were all involved in the NFκB pathway. ChIP-seq analyses obtained from the Cistrome database indicated that NFκB-p65 is likely to control the transcription of *CXCL10*, *TNFAIP3*, *INHBA*, and *PLAU*, and considering *TNFAIP3* and *PLAU* are the most significantly differentially expressed genes, we used chromatin immunoprecipitation-polymerase chain reaction (ChIP-PCR) to verify the regulation of p65 on their expression. The results demonstrated that SFE suppresses ESCC progression by down-regulating *TNFAIP3* and *PLAU* expression in a p65-dependent manner.

## 1. Introduction

Esophageal cancer is the sixth leading cause of cancer-related deaths worldwide, with many causes that vary by histologic type. Esophageal squamous cell carcinoma (ESCC) and adenocarcinoma (EADC) are the major subtypes of esophageal cancer and are epidemiologically and pathologically distinct. ESCC accounts for 70% of esophageal cancer cases worldwide, and cigarette smoking, alcohol consumption, and low intake of fruit and vegetables are the primary risk factors for esophageal cancer [1,2]. As symptoms from an obstructing lesion may be noticeable only when the tumor has reached a relatively advanced stage, the general prognosis is poor, and the 5-year survival rate is only 10–15%. Multimodality treatments, including surgical resection, chemotherapy, and radiotherapy, are offered to ESCC patients [3]. However, approximately one third of these patients show only partial response to chemotherapy and chemoradiation, and even among responders of these pre-surgery adjuvant treatments, some may gradually develop resistance. Therefore, proposals of novel therapeutic strategies and more treatment options for patients are urgently needed.

Sulforaphene (SFE), an isothiocyanate isolated from radish seed [4,5], has attracted increasing attention for its strong anticarcinogenic activities in experimental models. SFE has reportedly regulated several signaling pathways involved in cell proliferation, invasion, metastasis, and apoptosis. It inhibited triple-negative breast cancer by activating the tumor suppressor *Egr1* [6], induced hepatocellular carcinoma cell death by repressing keratin 8 and promoting anoikis [7], and targeted the PI3K-Akt pathway to cause lung cancer cell apoptosis [8]. We previously emphasized that SFE could also block ESCC progression by suppressing stearoyl-CoA desaturase (*SCD*) and cadherin 3 (*CDH3*) expression and boosting the GADD45B-MAP2K3-p38-p53 positive feedback loop [9].

The nuclear factor kappa B (NFκB) family consists of dimeric transcription factors central to the regulation of genes related to inflammation, the immune response, cell differentiation, proliferation, and survival [10]. Many inflammatory cascades enhance NFκB-dependent transcription, which in turn promotes inflammatory programs. In brief, activation of these cascades leads to increased inhibitory kappa kinase activity and phosphorylation, ubiquitination and degradation of the inhibitory proteins IκBα and IκBβ, eliciting NFκB-p65 translocation to the nucleus and transcription of proinflammatory genes [11]. Regarding tumorigenesis, NFκB has context-dependent effects that lead to elevated tumorigenic inflammation and stimulate tumor initiation and progression [12,13,14,15]. *TNFAIP3* and *PLAU* have been proven to be the target genes of NFκB [16,17,18,19]. *TNFAIP3* was originally identified as a protector of cells from TNF-induced cytotoxicity and, most notably, a repressor of excessive inflammation [20,21]. However, some recent studies implied a paradoxical role for *TNFAIP3* outside the immune system, suggesting its contributory effects to the proliferation and metastasis of a variety of cancer cells [22,23,24,25]. The evaluation of genes differentially expressed in normal esophageal mucosa and ESCC also identified *TNFAIP3* as a candidate biomarker of dysplasia or invasive ESCC [26]. uPA encoded by *PLAU* is produced by cancer cells and/or surrounding stromal cells as a proenzyme and converted to an active form when secreted into the tumor microenvironment by binding to the uPA receptor. uPA is involved in cell invasion and metastasis and is correlated with a poor prognosis of patients with one of various malignant tumors, including colon, breast, and stomach tumors [27,28,29].

We have verified *SCD*, *CDH3*, *MAP2K3* and *GADD45B* as the targets of SFE in ESCC through microarray analyses [9]; however, changing the expression of these genes could not completely reverse the inhibitory effect of SFE on ESCC proliferation and metastasis, which suggests that there must be some other mediators to connect SFE with ESCC characteristics. Since confirming the mechanism by which SFE inhibits ESCC progression contributes to the application of this isothiocyanate as a chemotherapeutic agent, we attempted to investigate SFE regulation of ESCC progression more deeply. Previously, we were absorbed in identifying individual genes that exhibited differences between ESCC cells with or without SFE treatment. Although useful, it failed to detect biological processes that are distributed across an entire gene network and slight at the level of individual genes. Gene Set Enrichment Analysis (GSEA) can overcome this analytical flaw as it focuses on groups of genes that are defined based on published biological knowledge about signaling pathways, and share common location, regulation, or biological function. Reinterpreting the data of microarray analyses by GSEA, we found the significantly differentially expressed genes in SFE-treated EC109 and KYSE510 cells were enriched in the NFκB pathway. The Gene Expression Omnibus (GEO) database is an international public repository which hosts high-throughput gene expression data and other categories of functional genomic data. Two independent cohorts archived in the GEO database were processed to pick out genes influencing ESCC characteristics and being regulated by SFE. Based on the ChIP-seq data of NFκB-p65 in multiple cancer cells and samples from the Cistrome database and subsequent verification experiments, we guessed and acknowledged that SFE could block the transcription-promoting activity of the NFκB pathway to suppress ESCC development. In short, all our efforts were to prove that SFE is a promising chemotherapeutic agent that may be used to treat ESCC in the future.

## 2. Results

### 2.1. Identification of SFE-Regulated Molecular Signatures in ESCC

**Figure 1:**
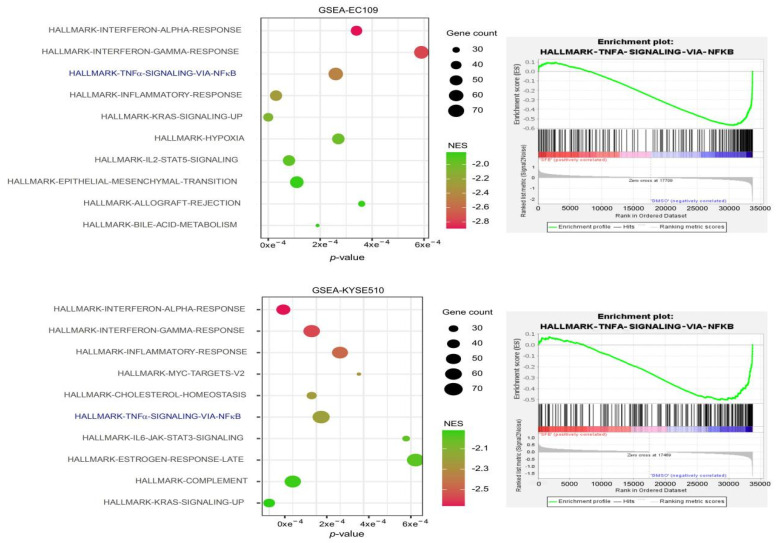
Enrichment of sulforaphene (SFE)-associated differentially expressed genes in esophageal squamous cell carcinoma (ESCC) cells. (**left**) The top 10 pathways of differentially expressed genes (DEGs) enriched in EC109 and KYSE510 cells. (**right**) The gene set enrichment analysis (GSEA) enrichment plot of tumour necrosis factor alpha-like (TNFα) Signaling via nuclear factor kappa B (NFκB) Pathway.

To shed more light on SFE-induced inhibition of ESCC tumorigenesis and development, we conducted GSEA to reanalyze previously described microarray results (GSE150891) [9] (Supplementary Figure S1A) and set the cutoff criteria as *p* < 0.05 and false discovery rate (FDR) < 0.05. We found that the significantly differentially expressed genes (DEGs) in ESCC cells treated with dimethyl sulfoxide (DMSO) as negative control were mainly enriched in pathways related to immunity, cell proliferation, and metastasis, such as epithelial mesenchymal transition, up-regulated KRAS proto-oncogene, GTPase (KRAS) signaling; IL6, Jak, and STAT3 signaling and IL2 and STAT5 signaling (Supplementary Figure S1B). The pathways meeting the screening criteria were sorted by normalized enrichment scores (NESs), and the top 10 in EC109 and KYSE510 cells are shown in Figure 1. We noticed that the TNFα_Signaling_via_the_NFκB_Pathway, which is closely related to caner progression, was identified in both ESCC cell lines.

**Figure 2:**
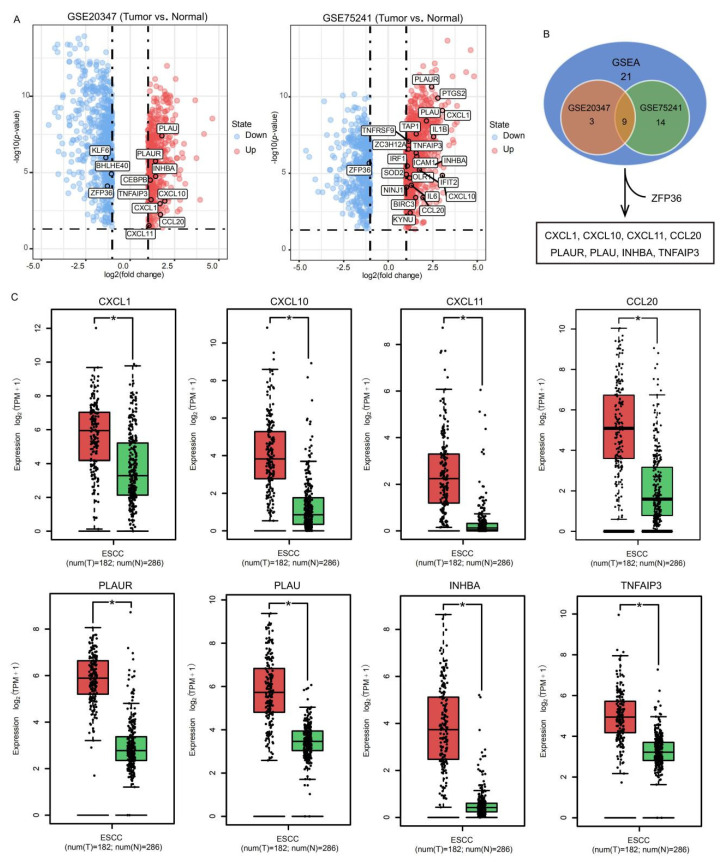
**Screening the potential target genes of SFE in ESCC cells.** (**A**) The volcano plots visualize the DEGs in GSE20347 and GSE75241. |log2(Fold Change)| > 1 and *p* < 0.05 were set as screening criteria. Genes overlapped with GSEA results are noted. (**B**) The common DEGs in GSEA results of EC109 and KYSE510, GSE20347, and GSE75241. (**C**) Comparison of the expression of C-X-C motif chemokine ligand 1 (*CXCL1*), C-X-C motif chemokine ligand 10 (*CXCL10*), C-X-C motif chemokine ligand 11 (*CXCL11*), C-C motif chemokine ligand 20 (*CCL20*), plasminogen activator, urokinase receptor (*PLAUR*), plasminogen activator, urokinase (*PLAU*), inhibin subunit beta A (*INHBA*)*,* and TNF alpha induced protein 3 (*TNFAIP3*) in ESCC and normal samples. The red and green boxes represent tumor (T) and normal (N) samples, respectively. The * represents *p* < 0.01.

**Figure 3:**
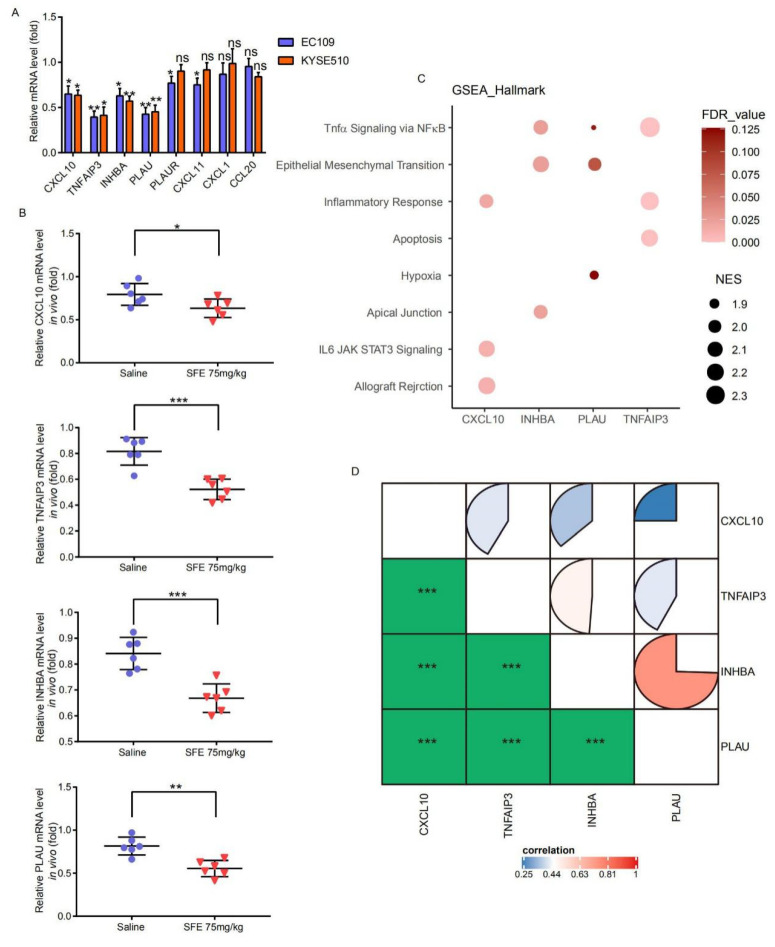
**Identifying the relation of SFE, *CXCL10*, *TNFAIP3*, *INHBA*, and *PLAU* in ESCC.** (**A**) The relative mRNA levels of *CXCL10*, *TNFAIP3*, *INHBA*, and *PLAU* in ESCC cells treated with SFE (20 μM). (**B**) The relative mRNA levels of *CXCL10*, *TNFAIP3*, *INHBA*, and *PLAU* in vivo. (**C**) The top 3 pathways of GSEA in high expression groups were gathered by bubble chart. (**D**) Gene expression correlation of *CXCL10*, *TNFAIP3*, *INHBA*, and *PLAU* in The Cancer Genome Atlas (TCGA) ESCC data. Data represent the mean ± SD of three independent experiments. NES, normalized enrichment score. The statistical significance was assessed by Student’s *t*-test. * *p* < 0.05, ** *p* < 0.01, and *** *p* < 0.005. ns, not significant.

We downloaded and processed the GSE20347 and GSE75241 datasets from the GEO database, which were obtained from the expression matrices of 17 and 15 paired ESCC samples and matched normal adjacent tissue samples, respectively, to acquire more ESCC development signatures. (Supplementary Figure S2). There were 12 DEGs in the GSE20347 dataset and 22 in the GSE75241 dataset that were also involved in the aforementioned TNFα_Signaling_via_the_NFκB_Pathway (Figure 2A). Excluding *ZFP36*, the change in the expression of which in the two datasets was inconsistent with that in the microarray analyses, we identified 8 genes as potential pivotal targets of SFE in ESCC (Figure 2B). The Cancer Genome Atlas (TCGA) is the largest and most commonly used public database, providing genomic sequence, expression, methylation, and copy number variation data on over 10,000 individuals which represent more than 30 different types of cancer, while the Genotype-Tissue Expression (GTEx) project established a data resource and tissue bank that release includes genotype data from 714 donors and over 11688 RNA-seq samples across 53 tissue sites. We downloaded the RNA-seq data of 182 ESCC samples and 13 normal tissues from the TCGA database, and 273 normal tissues from the GTEx database to detect the expression of these genes in tumor and normal samples. All these genes were overexpressed in the TCGA ESCC samples compared with in the normal tissues collected in the TCGA database or the TCGA and the GTEx databases (Supplementary Figure S3, Figure 2C). We then performed qRT-PCR in SFE-treated ESCC cells (Figure 3A) and ground tumor lumps (Figure 3B) from the previous xenograft tumor assay [9], verifying that the expression of *CXCL10*, *TNFAIP3*, *INHBA*, and *PLAU* was indeed controlled by SFE in ESCC.

### 2.2. Evaluation of the SFE-Regulated ESCC Progression Signatures in the TCGA Samples

We tried to use the RNA-seq data of 182 ESCC samples to further assess the biological characteristics of *CXCL10*, *TNFAIP3*, *INHBA*, and *PLAU*. The dataset was divided into a high-level group and a low-level group based on the median expression level of these 4 genes, and as shown in Supplementary Figure S4, all the high-level groups correlated with advanced pathology T stage and pathologic stage (except for *TNFAIP3*). We performed Kyoto Encyclopedia of Genes and Genomes (KEGG) and Gene Ontology (GO) biological process, cellular component, and molecular function functional enrichment analyses and found that highly expressed *CXCL10*, *TNFAIP3*, *INHBA*, and *PLAU* were positively correlated with the immune response, cell adhesion and growth (Figure S5A–D). The top 3 enriched pathways of these four genes in the ESCC samples are shown in Figure 3C, which shows a significant association between TNFα signaling via NFκB and high levels of *TNFAIP3*, *INHBA*, and *PLAU*. Although TNFα signaling via NFκB was not in the top 3 *CXCL10*-related pathways, it was statistically significant with NES >1.5. Interestingly, we found that the expression levels of *CXCL10*, *TNFAIP3*, *INHBA*, and *PLAU* were significantly correlated with each other in the TCGA ESCC samples (Figure 3D), and considering that p65 of the NFκB family can regulate genes involved in the immune response, cell proliferation, differentiation, and metastasis as a transcription factor, we speculated that these 4 genes might be the direct downstream targets of p65 in ESCC.

### 2.3. NFκB-p65 Can Induce TNFAIP3 and PLAU Expression in ESCC Cells

We searched publicly available ChIP-seq data related to *CXCL10*, *TNFAIP3*, *INHBA*, and *PLAU* in the Cistrome database to verify the association between p65 and its 4 possible targets. Supplementary Figure S6A shows the top 20 transcription factors that were the most likely regulators of *CXCL10*, *TNFAIP3*, *INHBA*, and *PLAU* based on the positions of transcription factor ChIP-seq peaks relative to the transcription start site. We found that p65 (corresponding to RELA proto-oncogene (*RELA*), the gene encoding p65, in Supplementary Figure S6A) can obviously regulate *CXCL10* and *TNFAIP3* expression, with the greatest potential for *TNFAIP3* regulation. To further analyze the possible promotion of p65 on gene expression, we selected ChIP-seq analyses of p65 in multiple cancer cells and samples (A549, HeLa, Huh7, LNCaP, MCF-7, and LoVo cells, and GM12892, GM15510, GM18505, GM18526, GM19099, and GM19193 datasets) that met all the quality criteria from the Cistrome database and visualized sample batches with the University of California Santa Cruz (UCSC) Genome Browser assembled for the human genome, GRCh38/hg38. These data indicated that p65 can directly bind to cis-regulatory elements and regulate the transcription rates of *CXCL10*, *TNFAIP3*, *INHBA*, and *PLAU* in diverse cancer cells (Supplementary Figure S6B–E). As H3K4me1 and H3K4me3 mark enhancers and promoters, respectively, and H3K27ac is associated with the active state of both elements, histone ChIP-seq data of 6 common cell lines (H1-hESC, HSMM, HUVEC, K562, NHEK, and NHLF cells) and esophagus muscularis mucosa samples from the Encyclopedia of DNA Elements (ENCODE) database were also aligned to the GRCh38/hg38 reference assembly to confirm the characteristics of p65-binding sites. We found that p65 can bind to the promoter regions of *CXCL10*, *TNFAIP3*, *INHBA*, and *PLAU* and the second intron region of *TNFAIP3*, as reported previously [16].

**Figure 4:**
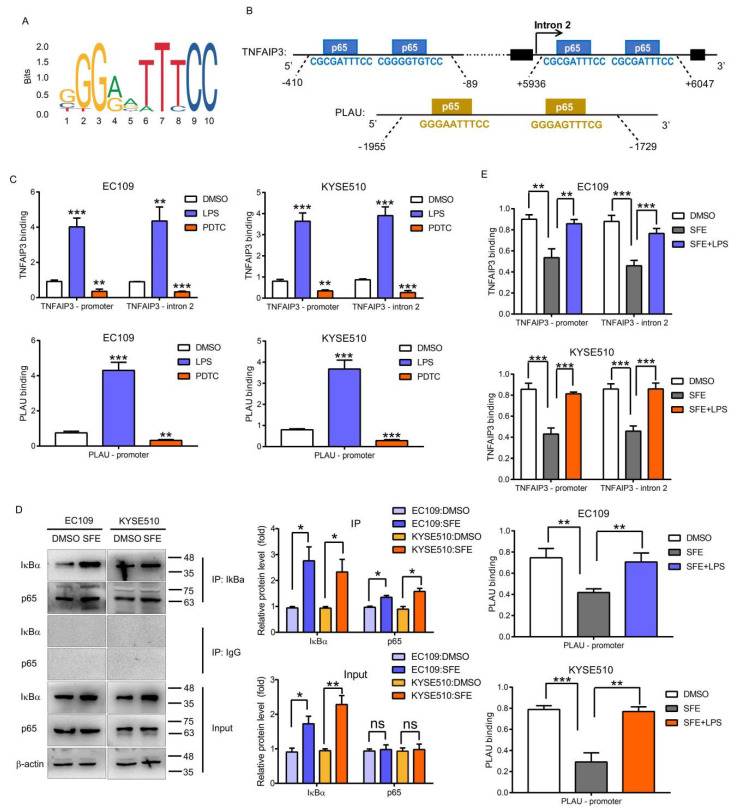
**The inhibitory effect of SFE on *TNFAIP3* and *PLAU* expression is NFκB-P65-dependent.** (**A**) NFκΒ−p65 binding logo collected in the JASPAR database. (**B**) Schematic diagram of the *TNFAIP3* and *PLAU* locus. The sequence and relative location of p65 binding sites are noted. (**C**) chromatin immunoprecipitation-polymerase chain reaction (ChIP-PCR) analysis of p65 occupancy within the *TNFAIP3* and *PLAU* loci in ESCC cells treated with DMSO, lipopolysaccharide (LPS) (0.3 mg/L), or ammonium pyrrolidinedithiocarbamate (PDTC) (80 μM) for 48 h. ChIP experiments were conducted in biological triplicate. (**D**) Co-immunoprecipitation (Co-IP) assays were carried out in EC109 and KYSE510 cells treated with SFE (20 μM) for 48 h. (**E**) ChIP-PCR analysis of p65 occupancy within the *TNFAIP3* and *PLAU* loci in ESCC cells treated with DMSO, SFE (20 μM), or a combination of SFE and LPS (0.3 mg/L) for 48 h. ChIP experiments were conducted in biological triplicate. Data represent the mean ± SD of three independent experiments. IP, immunoprecipitation. IgG, immunoglobulin G. The statistical significance was assessed by Student’s *t*-test. * *p* < 0.05, ** *p* < 0.01, and *** *p* < 0.005. ns, not significant.

We then tested whether p65 functions as a transcription factor to drive the expression of SFE targets in ESCC cells. The JASPAR database provides the sequence logo of p65 (Figure 4A) and predicts the binding sites in *CXCL10*, *TNFAIP3*, *INHBA*, and *PLAU*, and since *TNFAIP3* and *PLAU* were the most down-regulated genes in the SFE-treated ESCC cells, we chose these genes for follow-up verification (Figure 4B). lipopolysaccharide (LPS) [30] and ammonium pyrrolidinedithiocarbamate (PDTC) [31] are widely used agonists and inhibitors of the NFκB pathway, and we performed ChIP-PCR with an anti-p65 antibody in LPS- or PDTC-treated ESCC cells, confirming p65 occupancy at the predicted loci (Figure 4C). Additionally, the mRNA and protein levels of *TNFAIP3* and *PLAU* were changed after treating ESCC cells with LPS or PDTC (Supplementary Figure S7A). After verifying that SFE could increase the content of p65 bound to IκBα and inhibit p65 from entering the nuclear to inactivate the NFκB pathway (Figure 4D, Supplementary Figure S7B), we further identified the inhibitory effect of SFE on p65 binding to *TNFAIP3* and *PLAU*, which can be reversed by LPS treatment, analyzed by ChIP-PCR and luciferase reporter assays (Figure 4E, Supplementary Figure S8A). Also, the change trend of *TNFAIP3* and *PLAU* expression was consistent with that shown by the ChIP-PCR results (Supplementary Figure S8B). In summary, these results revealed that SFE prevented p65 from enhancing transcription to regulate *TNFAIP3* and *PLAU* expression.

### 2.4. SFE Suppresses TNFAIP3 and PLAU Expression by Inactivating the NFκB Pathway to Inhibit ESCC Cell Progression

**Figure 5:**
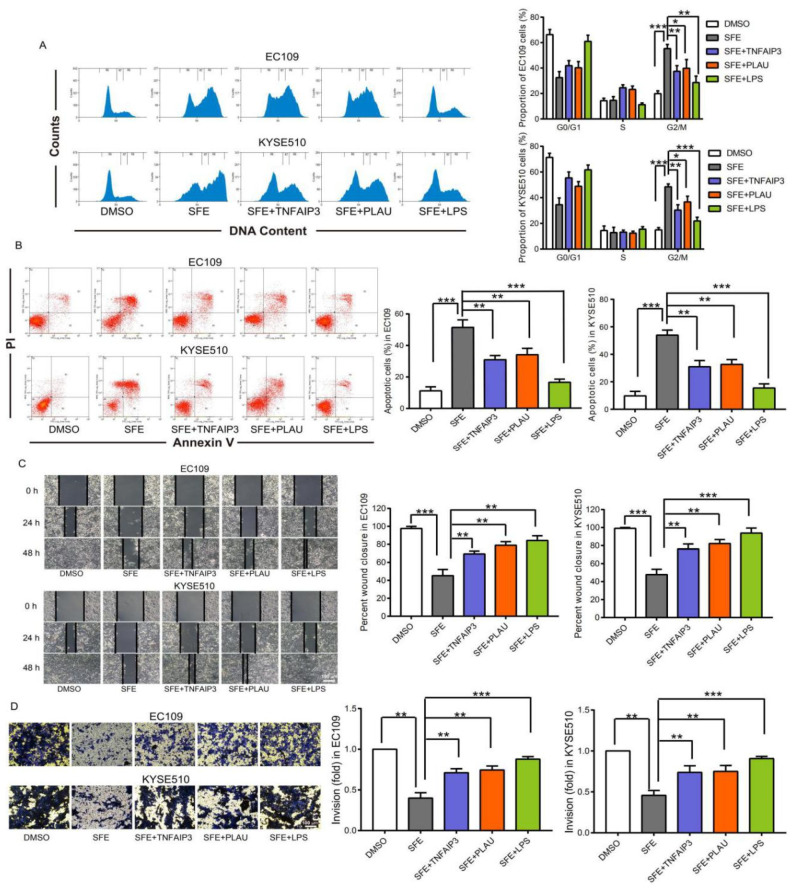
**SFE inhibits ESCC progression through suppressing *TNFAIP3* and *PLAU* expression in a NFκB-dependent manner.** (**A**) ESCC cells were treated with SFE (20 μM), SFE + pcDNA3.0-TNFAIP3 (SFE + TNFAIP3), SFE + pcDNA3.0 (SFE + PLAU), or SFE + LPS (0.3 mg/L) for 24 h, respectively, followed by assessing cell cycle distribution. (**B**) ESCC cells were treated with SFE (20 μM), SFE + pcDNA3.0-TNFAIP3 (SFE + TNFAIP3), SFE + pcDNA3.0 (SFE + PLAU), or SFE + LPS (0.3 mg/L) for 48 h, respectively, followed by assessing the apoptotic rates. (**C**,**D**) Scrape motility assay (**C**) and trans-well assay (**D**) were performed in ESCC cells with SFE (20 μM), SFE + pcDNA3.0-TNFAIP3 (SFE + TNFAIP3), SFE + pcDNA3.0 (SFE + PLAU), or SFE + LPS (0.3 mg/L) treatment. Data represent the mean ± SD of three independent experiments. The statistical significance was assessed by Student’s *t*-test. * *p* < 0.05, ** *p* < 0.01, and *** *p* < 0.005.

We next sought to determine whether *TNFAIP3*, *PLAU*, and their regulator p65 were involved in SFE-mediated inhibition of ESCC cell progression. We previously confirmed that SFE induced ESCC cell G2/M arrest and apoptosis to inhibit cell proliferation, and reduced the invasive and migratory abilities of ESCC cells [9]. Flow cytometry analyses, scrape motility, and trans-well assays shown in Figure 5 also suggested SFE inhibition of ESCC cell progression. However, when overexpressing *TNFAIP3* and *PLAU* (Supplementary Figure S9A,B) or upon activation of the NFκB pathway, all the inhibitory effects of SFE were reversed (Figure 5A–D), suggesting that SFE blocked the p65 promotion of *TNFAIP3* and *PLAU* expression to inhibit ESCC cell proliferation and metastasis.

## 3. Discussion

ESCC is the most common type of esophageal cancer, a serious malignancy considering both the mortality and prognosis associated with it, and accounts for 70% of all ESCC cases. Owing to recurrence, diagnosis in advanced stages, extensive invasion, and metastasis, the overall 5-year survival of ESCC worldwide is lower than 15% after the initial diagnosis. Therefore, it is of utmost urgency to further comprehend the molecular mechanisms that underlie the initiation, development, and metastasis of ESCC. To improve the prognosis and reduce the mortality of patients, identifying novel therapeutic targets and effective diagnostic markers should be on the research agenda. As an isothiocyanate isolated from radish seeds, SFE has attracted increasing attention for its anticancer effects in multiple cancers, such as breast cancer, lung cancer, ovarian cancer, and hepatocellular carcinoma. We also verified the inhibitory influence of SFE on ESCC cell proliferation and metastasis, providing strong proof for the broad-spectrum anticarcinogenic activity of SFE. In this study, we attempted to further shed light on the mechanism involved in the regulation of SFE on ESCC cell progression with the assistance of various bioinformatics analyses.

We reanalyzed the microarray results of SFE-treated ESCC cells with GSEA and identified the DEGs that are enriched in TNFα_Signaling_via_the_NFκB_Pathway. After screening or the shared genes in the GSE20374 and GSE75241 datasets with the aforementioned NFκB pathway, we confirmed 8 genes that were most likely to be involved in SFE inhibition of ESCC progression, 4 of which were eventually identified as the targets of SFE in ESCC. We then downloaded the RNA-seq analysis results obtained from ESCC samples in the TCGA database and categorized all the data into two phenotype groups, the high-level group and low-level group, according to the median expression level of *CXCL10*, *TNFAIP3*, *INHBA*, and *PLAU*, respectively. GSEA was applied to perform KEGG and GO biological process, cellular component, and molecular function functional enrichment analyses with *CXCL10*, *TNFAIP3*, *INHBA*, and *PLAU* in ESCC samples. We found that the high levels of all 4 genes were related to the immune response and cell adhesion and growth and were significantly and positively correlated with the NFκB pathway, which was consistent with the microarray analysis of ESCC cells. While processing RNA-seq data of *CXCL10*, *TNFAIP3*, *INHBA*, and *PLAU* in ESCC samples, we noticed that the expression levels of these genes were correlated with each other, and as the p65 in the NFκB family is an established transcription factor, we hypothesized that these 4 genes might be directly regulated by p65 in ESCC. We searched publicly available ChIP-seq results associated with p65 and aligned the data to the GRCh38/hg38 reference assembly using UCSC Browser, finding that p65 can bind to the promoter regions of *CXCL10*, *TNFAIP3*, *INHBA*, and *PLAU* and the second intron region of *TNFAIP3* in various kinds of cancer cells and samples. We selected *TNFAIP3* and *PLAU* to verify the regulation of p65 on the expression of these two genes because they were down-regulated to the greatest extent in the SFE-treated ESCC cells. The ChIP-PCR results indicated that p65 functions as a transcription factor to regulate the transcription rates of *TNFAIP3* and *PLAU*, and SFE can block this effect. After confirming that SFE inactivated the NFκB pathway and decreased the content of p65 in the nuclear, we can conclude that SFE prevents p65 from functioning as a regulator of gene expression and resulting in the down-regulation of *TNFAIP3* and *PLAU*. The dose-dependent inhibition of SFE on ESCC cell proliferation, invasion and migration had been confirmed previously [9], and data shown in Figure 5 could also indicate that SFE induced G2/M cell cycle arrest, cell apoptosis, and decreased the metastasis of ESCC cells. However, when overexpressing *TNFAIP3* and *PLAU*, or activating the NFκB pathway by LPS, the proliferative and metastatic abilities of SFE-treated cells were partially restored. These results suggested that SFE suppressed ESCC progression through inactivating the NFκB pathway which lowered *TNFAIP3* and *PLAU* expression. As far as we know, this is the first study to verify *TNFAIP3* and *PLAU* as direct downstream targets of p65 in ESCC and to reveal the association between the NFκB pathway and SFE suppression of ESCC progression. Of course, further studies are warranted to investigate the regulatory mechanism of SFE on ESCC development. The GO enrichment analysis results of the high-level ESCC sample group showed that these genes are related to extracellular matrix, which is benefits to tumor proliferation, invasion and migration [32,33], suggesting that high levels of *CXCL10*, *TNFAIP3*, *INHBA*, and *PLAU* might affect the structure and composition of the extracellular matrix to promote ESCC progression. Therefore, next, we will focus on whether SFE has an inhibitory effect on extracellular matrix formation and maintenance. Additionally, the positive influence of p65 on *CXCL10* and *INHBA* expression in ESCC cells needs to be confirmed in the future.

In summary, we identified that SFE inactivated the NFκB pathway and down-regulated *TNFAIP3* and *PLAU* expression to suppress ESCC cell proliferation and metastasis, providing new insights into the anticarcinogenic activity of SFE and confirming SFE as a potential chemotherapeutic agent.

## 4. Materials and Methods

### 4.1. Cell Culture and Chemicals

The human esophageal cancer EC109, KYSE510 cell lines were obtained from the National Infrastructure of Cell Line Resource, cultured in RPMI-1640 medium (Gibco, Grand Island, NY, USA) with 10% fetal bovine serum (FBS) (Gibco), 100 units/mL penicillin (Invitrogen, Carlsbad, CA, USA) and 100 mg/mL streptomycin (Invitrogen). The cell lines were characterized by Genetic Testing Biotechnology Corporation (Suzhou, China) using short tandem repeat markers within the last three years and they were not contaminated by mycoplasma detected by Myco-Lumi Luminescent Mycoplasma Detection Kit (Beyotime, Shanghai, China). SFE was separated and purified from radish seeds (Beijing Tongrentang Co., LTD, Beijing, China) as reported previously [4,5], dissolved in DMSO (Beijing Chemical Factory, Beijing, China). LPS (HY-D1056) and PDTC (HY-18738) was obtained from MedChem Express (Monmouth Junction, New Jersey, USA).

### 4.2. Plasmid Construction

Full-length homo sapiens *TNFAIP3* and *PLAU* were cloned into the pcDNA3.0 (pC3.0) plasmid, producing pC3.0-TNFAIP3 and pC3.0-PLAU plasmids. All the plasmids were purchased from Genepharma (Shanghai, China).

### 4.3. Cell Apoptosis and Cell Cycle Analysis

Cells treated with DMAO, SFE (20 μΜ) and transfected with plasmiads or treated with LPS (0.3 mg/L) in combination with SFE treatment, were collected after 24 h for cell cycle analysis and 48 h for cell apoptosis analysis. Cell apoptosis was determined using the Dead Cell Apoptosis Kit with Annexin V Alexa Fluor™ 488 and Propidium Iodide (Invitrogen) and cell cycle distribution was analyzed with Cell Cycle Detection Kit (KeyGEN BioTECH, Jiangsu, China) according to the manufacturer’s instructions. Both analyses were detected with MoFlo XDP flow cytometer (Beckman Coulter, Miami, FL, USA) and data was processed by Summit V5.2.1 (Beckman Coulter).

### 4.4. Scrape Motility and Trans-Well Assays

In scrape motility assay, cells were scratched with a sterile 100 µl pipette tip and photographed at × 100 magnification using BEION medical image software V4.20 (Beion, Shanghai, China) at different time points. In trans-well assay, the trans-well chambers (Corning, NY, USA) were covered with matrigel (Biosciences, San Jose, CA, USA) overnight. Cells cultured in 1 % FBS were added to the chambers and medium with 10 % FBS was added to the lower wells. After 48 h incubation, the number of cells invading through the matrigel was counted in 6 randomly selected visual fields using a Leica DM3000 microscope (Leica, Wetzlar, Germany). Data was analyzed by ImageJ 2X software.

### 4.5. Quantitative Reverse Transcription PCR (qRT-PCR)

Total RNA was extracted from cells or grinded tumor lumps treated with trizol reagent (Invitrogen). Each sample was reverse transcribed into cDNA with the PrimeScript™RT Master Mix (TaKaRa). SYBR Green Real-time PCR Master Mix (TOYOBO, Osaka, Japan) and ABI 7500 real-time PCR system (Applied Biosystems) were used to measure the expression of target genes according to the recommendations of the manufacturer. Gene expression was calculated relative to β-actin, an internal reference gene, using the 2method. Primers were shown in Supplementary Table S1.

### 4.6. Nuclear and Cytoplasmic Protein Extraction

Extraction was performed using Nuclear and Cytoplasmic Protein Extraction Kit (Beyotime). Briefly, cells were resuspended in cytoplasmic protein isolation solution A with phenylmethanesulfonyl fluoride (PMSF) (Beyotime). Next, homogenate was treated with cytoplasmic protein isolation solution B and centrifuged at 4 °C for 10 min. The obtained supernatant was cytoplasmic protein fraction. Then the precipitate was resuspended in nuclear protein isolation solution with PMSF, vortexed and homogenized on ice alternately for 30 min and centrifuged at 4 °C for 10 min. The supernatant was nuclear protein fraction.

### 4.7. Western Blotting Assay

Protein was isolated from cells or grinded tumor lumps using Radio-Immunoprecipitation Assay (RIPA) Lysis Buffer (Beyotime) with PMSF. After measuring protein concentration by Bicinchonininc Acid (BCA) Protein Assay Kit (Beyotime), all the samples were boiled with 4 x SDS-PAGE Sample Loading Buffer (Beyotime) for 7 min at 100 °C. Then protein was separated by SDS-PAGE and transferred to polyvinylidene fluoride (PVDF) membranes (Millipore, Darmstadt, Germany). Membranes were blocked by 5% milk and immunoblotted with primary antibodies (Supplementary Table S2). After incubation with HRP-labeled goat anti-mouse immunoglobulin G (IgG) or goat anti-rabbit IgG (Beyotime), the blots were detected using the Chemiluminescence Image Analysis System (Tanon, Shanghai, China) with Enhanced Chemiluminescence (ECL) Luminescence reagent (Sangon Biotech, Shanghai, China). β-actin and lamin B1 were used as loading control.

### 4.8. Chromatin Immunoprecipitation (ChIP) and ChIP-qPCR Assays

Chromatin immunoprecipitation (ChIP) was performed using a Magna ChIP™ Protein G Magnetic Beads (Millipore, MA, USA) (16-662) according to the manufacturer’s instructions. EC109 cells were cultured in 100-mm dishes and treated with DMSO, SFE (20 μΜ), or with a combination of SFE and LPS (0.3 mg/L) for 48 h. After fixed with 1% formaldehyde for 10 min and washed with cold phosphate buffer saline (PBS), cells were lysed using cell and nuclear lysis buffer and sonicated on ice using a Sonics Vibra-Cell processor (Sonics & Materials Inc., Newtown, CT, United Kingdom) to generate DNA fragments. Approximately 2% of the suspension was removed to determine the input quantity of DNA. Then chromatin was immunoprecipitated by incubating overnight at 4 °C with protein G magnetic beads and the following antibodies: 3 μg rabbit anti-NF-κB p65 (8242, Cell Signaling Technology) and normal rabbit IgG (2729, Cell Signaling Technology). The precipitated DNA-protein complexes were washed with wash buffer and eluted in elution buffer. RNA was digested with RNase A for 30 min at 37 °C, and proteins were digested with Proteinase K for 2 h at 45 °C. After DNA purification, the resulting DNA was analyzed by qPCR and normalized by total chromatin (input). Primers specific to the predicted binding sites of p65 in *TNFAIP3* and *PLAU* are described in Supplementary Table S1.

### 4.9. Co-Immunoprecipitation (Co-IP) Assay

Co-IP was performed as previously described [9]. Briefly, lysate of EC109 and KYSE510 cells treated with SFE (20 µM) for 48 h were generated under addition of Halt Protease Inhibitor Cocktail (Thermo Fisher Scientific, Waltham, MA, USA) and Halt Phosphatase Inhibitor Cocktail (Thermo Fisher Scientific). Protein concentration was measured by the Pierce BCA Protein Assay Kit (Thermo Fisher Scientific). A total of 2250 µg/mL protein was used for co-IP assay performed with the Pierce™ Co-Immunoprecipitation Kit (Thermo Fisher Scientific). 35 µg of the IκBα primary antibody was incubated with the delivered resin and covalently coupled for 2 h. The antibody-coupled resin was incubated with 200 µL cell lysates overnight at 4 °C, and then the protein complexes were eluted. Subsequent western blotting assay was performed as described before.

### 4.10. Luciferase Reporter Assay

The predicted binding sites of p65 in *TNFAIP3* and *PLAU* promoters and the intron 2 of *TNFAIP3* were cloned into pRP-Puro-Luc vector (Cyagen, Santa Clara, CA, USA) to construct plasmids named TNFAIP3-promoter-Luc, PLAU-promoter-Luc, and TNFAIP3-intron 2-Luc. These plasmids were co-transfected with pRL-TK (Promega, Madison, WI, USA) expressing renilla luciferase as the internal control into EC109 and KYSE510 cells, followed by LPS (0.3 mg/L), SFE (20 µM) or a combination of SFE and LPS treatment for 48 h. Then the luciferase activity was measured by the Dual Luciferase Reporter Gene Assay Kit (Beyotime). The relative activity was calculated by normalizing to the renilla luciferase activity.

### 4.11. Statistics and Bioinformatics

GSEA v2.0 [34] was used to determine enrichment of significantly DEGs in microarray data of EC109 and KYSE510 cells treated with 20 μM SFE, and the high mRNA levels of *CXCL10*, *TNFAIP3*, *INHBA*, and *PLAU*-related GO annotations and pathways in Illumina HiSeq 2000 RNA Sequencing of TCGA ESCC data. RNA-seq data was separated into two phenotypes for GSEA according to the median of *CXCL10*, *TNFAIP3*, *INHBA*, and *PLAU* expression respectively: high-level group and low-level group. The expression of genes was submitted to GSEA V2.0, using the hallmark gene sets from The Molecular Signatures Database (MSigDB) (https://www.gsea-msigdb.org/gsea/msigdb/index.jsp) and log2 ratio of classes method. Multiple probe matches for the same gene were collapsed into one value and the highest probe reading was used in each case. GSEA was run with a weighted statistic and evaluated by comparison to results obtained using 500 random permutations of each data set. Default settings were used for all other parameter. The significant enrichment results were demonstrated based on NES, *p*-value, and FDR value.

GSE20347 [35] and GSE75241 [36] were analyzed using the Sangerbox tools (http://www.sangerbox.com/tool) to identify genes that overlap with the GSEA enrichment analysis of EC109 and KYSE510 microarray data (GSE150891).

RNA-seq data collected in the TCGA and GTEx databases were downloaded from the UCSC Xena (https://xenabrowser.net/datapages/). Excluding duplicate samples, metastatic samples, and samples with incomplete clinical information, we got a final dataset of 182 primary ESCC samples and 13 normal samples (from muscularis, mucosa, gastroesophageal junction) in the TCGA database, and 273 normal samples (from muscularis, mucosa, gastroesophageal junction) in the GTEx database.

The JASPAR CORE database (http://jaspar.genereg.net/) was selected to determine the sequence logo of NFκB-p65 protein, and predict the binding sites in *TNFAIP3* and *PLAU*. ChIP-seq data of p65 with multiple biological sources is displayed in Cistrome Data Browser (http://cistrome.org/db/#/), and samples passed all the quality controls (A549, Hela, Huh7, LNCaP, MCF-7, LoVo, GM12892, GM15510, GM18505, GM18526, GM19099, GM19193) were aligned to the GRCh38/hg38 reference assembly using UCSC Browser. To further comprehend the information of p65 binding sites in *TNFAIP3* and *PLAU*, histone ChIP-seq data of 6 cell lines (H1-hESC, HSMM, HUVEC, K562, NHEK, NHLF) and esophagus muscularis mucosa samples collected by the ENCODE database were also visualized by UCSC Browser and all experiments were conducted three times

Data was analyzed and graphs were plotted by Prism software version 7 (GraphPad software Inc., San Diego, CA, USA). For association between gene expression and pathologic stage or pathology Tumor-Node-Metastasis (TNM) stage, data was analyzed via Kruskal–Wallis test. For gene expression in ESCC samples from TCGA and GTEx database, data was analyzed via one-way analysis of variance (ANOVA). For other statistical analyses, results were represented as mean ± SD of three independent experiments and significance was determined using the two-tailed Student’s *t*-test. *p* < 0.05 and FDR < 0.05 was considered statistically significant for GSEA of microarray data in SFE-treated ESCC cells, GSE20347, and GSE75241 datasets, and statistical significance was considered starting from *p* < 0.05 and FDR < 0.25 for KEGG and GO functional enrichment analyses of *CXCL10*, *TNFAIP3*, *INHBA*, and *PLAU*. For other analyses, differences were considered to be significant at *p* < 0.05.

## Data Availability

Publicly available datasets were analyzed in this study. These data can be found here: MSigDB, https://www.gsea-msigdb.org/gsea/msigdb/index.jsp; GSE150891, GSE20347, and GSE75241, https://www.ncbi.nlm.nih.gov/geo/; UCSC Xena, https://xenabrowser.net/datapages/; JASPAR CORE database, http://jaspar.genereg.net/; Cistrome Data Browser, http://cistrome.org/db/#/.

## Supplementary Materials

Supplementary materials can be found at https://www.mdpi.com/1422-0067/22/1/60/s1. Figure S1: Enrichment of SFE-associated differentially expressed genes in ESCC cells, Figure S2: Analysis of GSE20347 and GSE75241 dataset, Figure S3. *CXCL1, CXCL10, CXCL11, CCL20, PLAUR, PLAU, INHBA, TNFAIP3* are overexpressed in the ESCC samples (*n* = 182) compared with the normal samples (*n* = 13) collected in the TCGA database. Figure S4: Correlation between the expression of *CXCL10*, *TNFAIP3*, *INHBA*, *PLAU*, and the pathologic stage, pathology T stage of ESCC patients, Figure S5: GO analyses of *CXCL10, TNFAIP3, INHBA, and PLAU*, Figure S6: NFκB p65 regulates the transcription of *CXCL10, TNFAIP3, INHBA*, and *PLAU*, Figure S7: SFE inactivates the NFκB pathway to reduce *TNFAIP3* and *PLAU* expression, Figure S8: SFE can inhibit NFκB from promoting the transcription of *TNFAIP3* and *PLAU*. Figure S9: The effect of pC3.0-TNFAIP3 and pC3.0-PLAU transfected on *TNFAIP3* and *PLAU* expression, Table S1: Primer sequence for qPCR and ChIP-PCR, Table S2: Primary antibodies used in this study.
