# Supplementary material for: Identifying the p65-Dependent Effect of Sulforaphene on Esophageal Squamous Cell Carcinoma Progression via Bioinformatics Analysis
Source: Int J Mol Sci. 2020 Dec 23;22(1):60. doi: 10.3390/ijms22010060 (PMC7793474; doi:10.3390/ijms22010060)
Supplement: Supplementary file 1 [file ijms-22-00060-s001.zip › supplementary figure_s1.PDF.pdf]

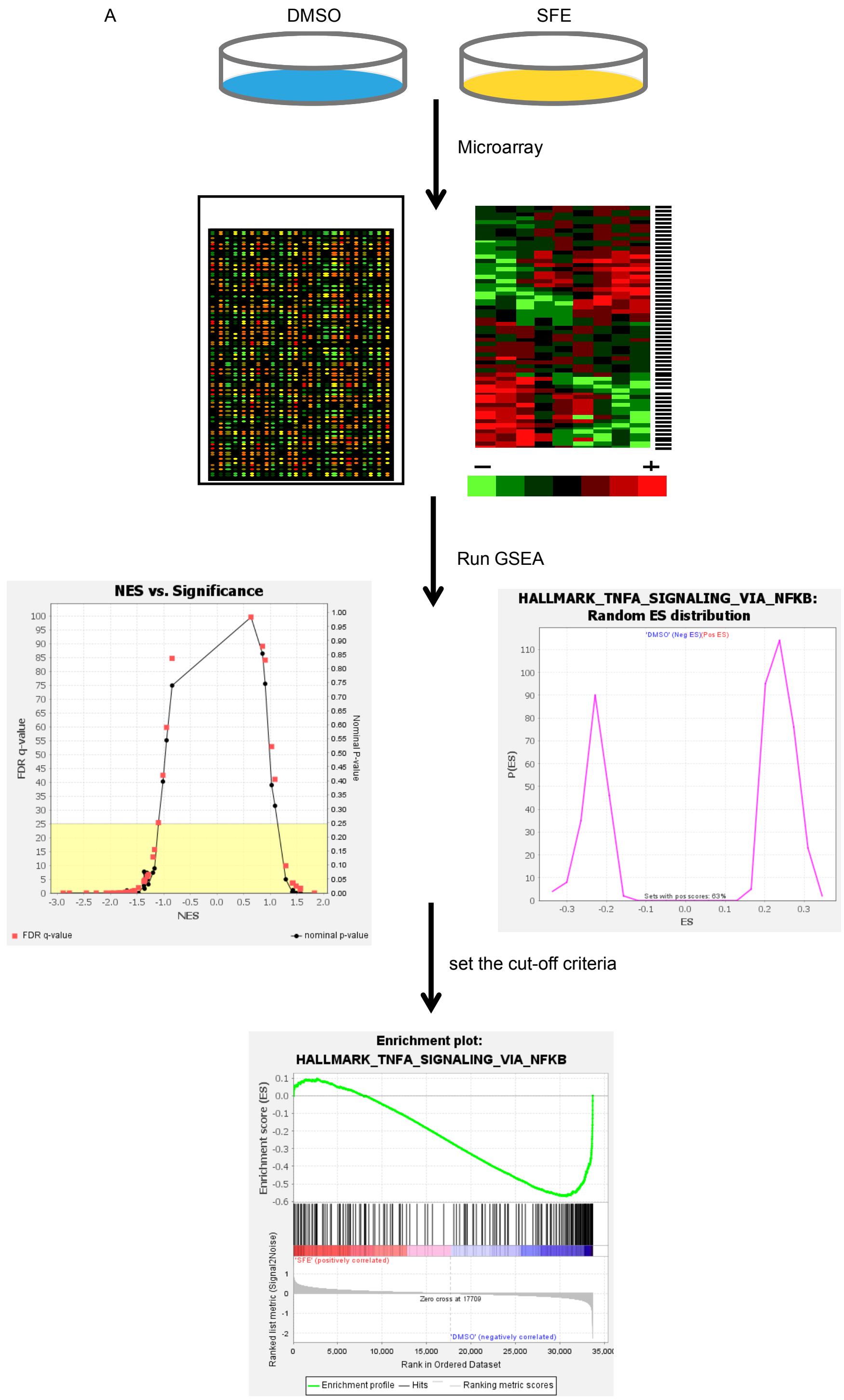
A
DMSO
SFE
Microarray
Run GSEA
NES vs. Significance
FDR q-value
Nominal P-value
NES
FDR q-value
nominal p-value
HALLMARK_TNFA_SIGNALING_VIA_NFKB: Random ES distribution
'DMSO' (Neg ES)(Pos ES)
Sets with pos scores: 63%
P(ES)
ES
set the cut-off criteria
Enrichment plot: HALLMARK_TNFA_SIGNALING_VIA_NFKB
Enrichment score (ES)
'SFE' (positively correlated)
Zero cross at 17709
'DMSO' (negatively correlated)
Ranked list metric (Signal2Noise)
Rank in Ordered Dataset
Enrichment profile
Hits
Ranking metric scores

B

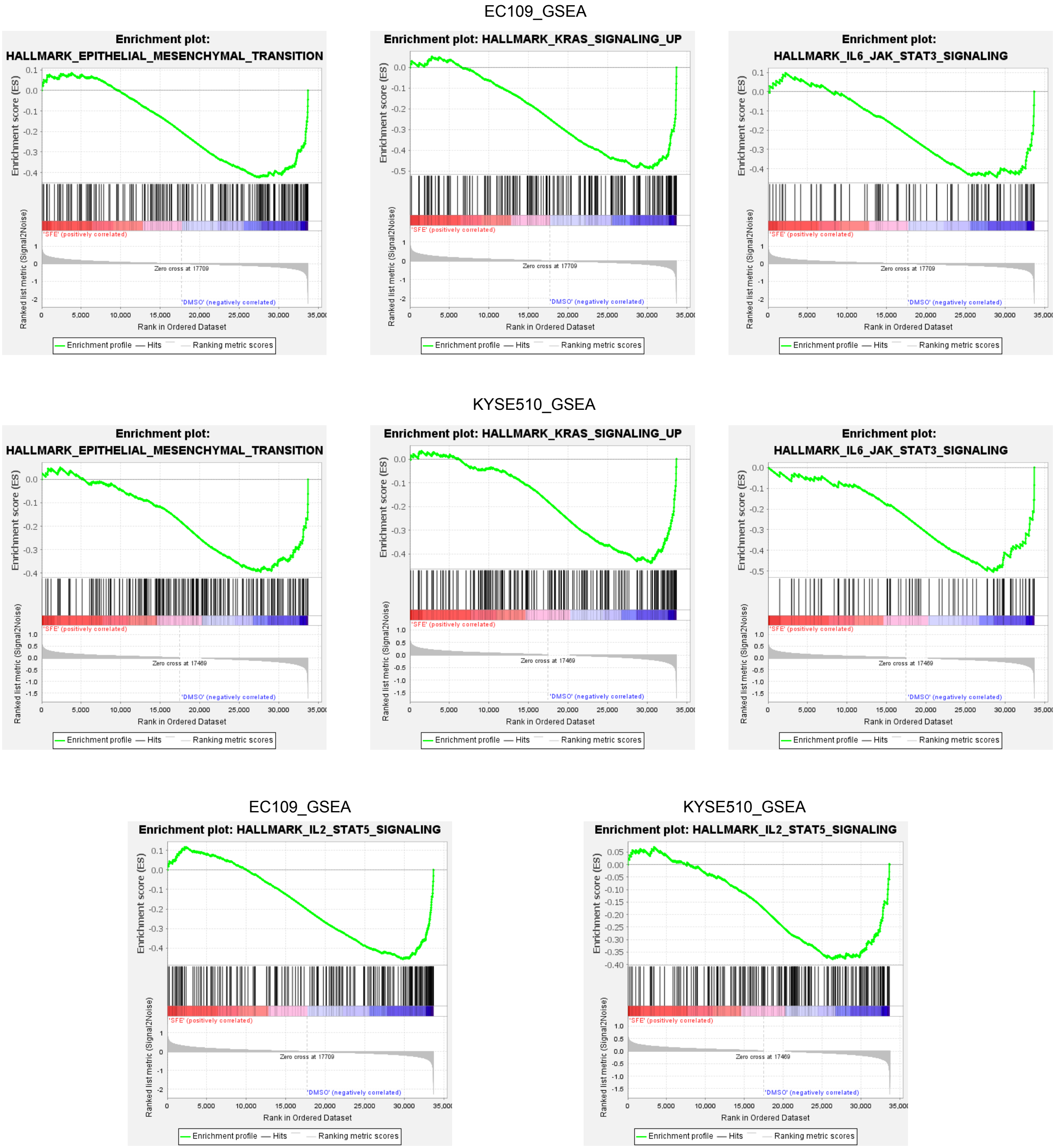

Figure s1. Enrichment of SFE-associated differentially expressed genes in ESCC cells. (A) Schematic overview of GSEA in ESCC cells with or without SFE-treatment. (B) The GSEA enrichment plot of Epithelial Mesenchymal Transition, KRAS Signaling Up, IL6 JAK STAT3 Signaling and IL2 STAT5 Signaling.
