# Supplementary material for: Identifying the p65-Dependent Effect of Sulforaphene on Esophageal Squamous Cell Carcinoma Progression via Bioinformatics Analysis
Source: Int J Mol Sci. 2020 Dec 23;22(1):60. doi: 10.3390/ijms22010060 (PMC7793474; doi:10.3390/ijms22010060)
Supplement: Supplementary file 1 [file ijms-22-00060-s001.zip › supplementary figure_s3.PDF.pdf]

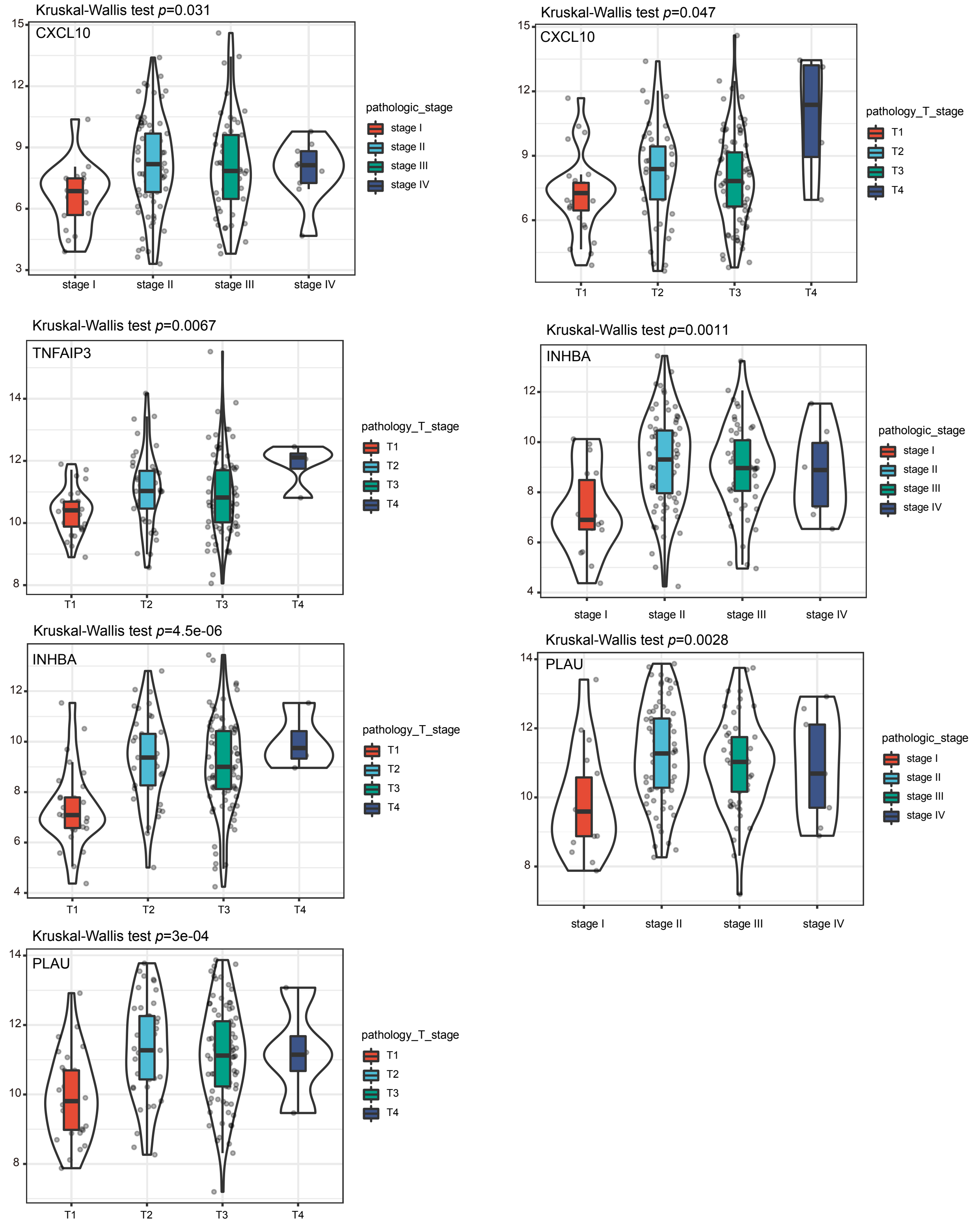

Figure s3. Correlation between the expression of *CXCL10, TNFAIP3, INHBA, PLAU* and the pathologic stage, pathology T stage of ESCC patients. Violin plots show the high levels of *CXCL10, TNFAIP3, INHBA* and *PLAU* are all positively correlated with pathologic stage (with the exception of *TNFAIP3* expression) and pathology T stage. The statistical significance was assessed by Kruskal-Wallis test.
