# Supplementary material for: Identifying the p65-Dependent Effect of Sulforaphene on Esophageal Squamous Cell Carcinoma Progression via Bioinformatics Analysis
Source: Int J Mol Sci. 2020 Dec 23;22(1):60. doi: 10.3390/ijms22010060 (PMC7793474; doi:10.3390/ijms22010060)
Supplement: Supplementary file 1 [file ijms-22-00060-s001.zip › supplementary figure_s4.PDF.pdf]

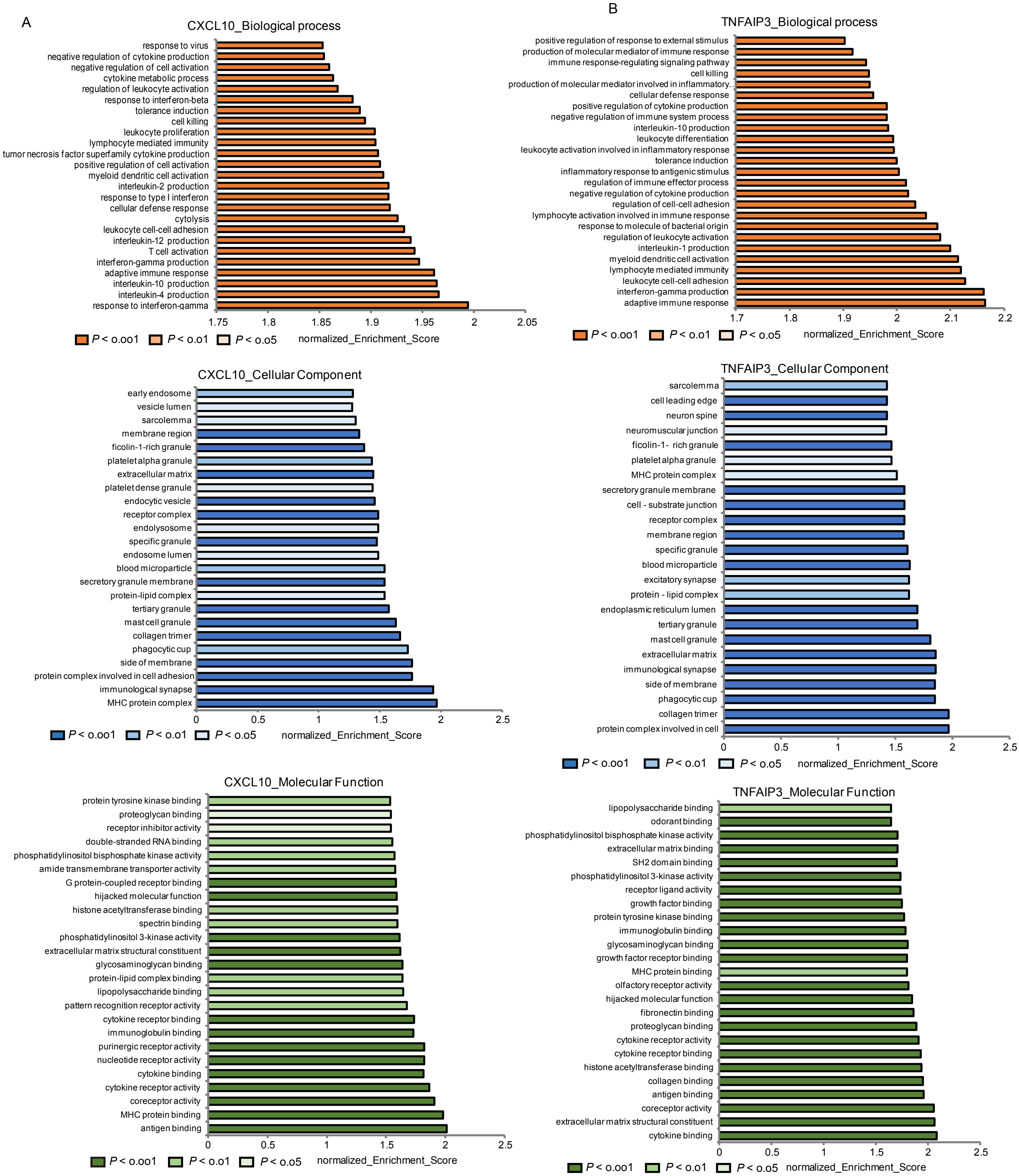
A
CXCL10_Biological process
response to virus
negative regulation of cytokine production
negative regulation of cell activation
cytokine metabolic process
regulation of leukocyte activation
response to interferon-beta
tolerance induction
cell killing
leukocyte proliferation
lymphocyte mediated immunity
tumor necrosis factor superfamily cytokine production
positive regulation of cell activation
myeloid dendritic cell activation
interleukin-2 production
response to type I interferon
cellular defense response
cytolysis
leukocyte cell-cell adhesion
interleukin-12 production
T cell activation
interferon-gamma production
adaptive immune response
interleukin-10 production
interleukin-4 production
response to interferon-gamma
1.75 1.8 1.85 1.9 1.95 2 2.05
P < 0.001 P < 0.01 P < 0.05 normalized_Enrichment_Score
B
TNFAIP3_Biological process
positive regulation of response to external stimulus
production of molecular mediator of immune response
immune response-regulating signaling pathway
cell killing
production of molecular mediator involved in inflammatory...
cellular defense response
positive regulation of cytokine production
negative regulation of immune system process
interleukin-10 production
leukocyte differentiation
leukocyte activation involved in inflammatory response
tolerance induction
inflammatory response to antigenic stimulus
regulation of immune effector process
negative regulation of cytokine production
regulation of cell-cell adhesion
lymphocyte activation involved in immune response
response to molecule of bacterial origin
regulation of leukocyte activation
interleukin-1 production
myeloid dendritic cell activation
lymphocyte mediated immunity
leukocyte cell-cell adhesion
interferon-gamma production
adaptive immune response
1.7 1.8 1.9 2 2.1 2.2
P < 0.001 P < 0.01 P < 0.05 normalized_Enrichment_Score
CXCL10_Cellular Component
early endosome
vesicle lumen
sarcolemma
membrane region
ficolin-1-rich granule
platelet alpha granule
extracellular matrix
platelet dense granule
endocytic vesicle
receptor complex
endolysosome
specific granule
endosome lumen
blood microparticle
secretory granule membrane
protein-lipid complex
tertiary granule
mast cell granule
collagen trimer
phagocytic cup
side of membrane
protein complex involved in cell adhesion
immunological synapse
MHC protein complex
0 0.5 1 1.5 2 2.5
P < 0.001 P < 0.01 P < 0.05 normalized_Enrichment_Score
TNFAIP3_Cellular Component
sarcolemma
cell leading edge
neuron spine
neuromuscular junction
ficolin-1- rich granule
platelet alpha granule
MHC protein complex
secretory granule membrane
cell - substrate junction
receptor complex
membrane region
specific granule
blood microparticle
excitatory synapse
protein - lipid complex
endoplasmic reticulum lumen
tertiary granule
mast cell granule
extracellular matrix
immunological synapse
side of membrane
phagocytic cup
collagen trimer
protein complex involved in cell
0 0.5 1 1.5 2 2.5
P < 0.001 P < 0.01 P < 0.05 normalized_Enrichment_Score
CXCL10_Molecular Function
protein tyrosine kinase binding
proteoglycan binding
receptor inhibitor activity
double-stranded RNA binding
phosphatidylinositol bisphosphate kinase activity
amide transmembrane transporter activity
G protein-coupled receptor binding
hijacked molecular function
histone acetyltransferase binding
spectrin binding
phosphatidylinositol 3-kinase activity
extracellular matrix structural constituent
glycosaminoglycan binding
protein-lipid complex binding
lipopolysaccharide binding
pattern recognition receptor activity
cytokine receptor binding
immunoglobulin binding
purinergic receptor activity
nucleotide receptor activity
cytokine binding
cytokine receptor activity
coreceptor activity
MHC protein binding
antigen binding
0 0.5 1 1.5 2 2.5
P < 0.001 P < 0.01 P < 0.05 normalized_Enrichment_Score
TNFAIP3_Molecular Function
lipopolysaccharide binding
odorant binding
phosphatidylinositol bisphosphate kinase activity
extracellular matrix binding
SH2 domain binding
phosphatidylinositol 3-kinase activity
receptor ligand activity
growth factor binding
protein tyrosine kinase binding
immunoglobulin binding
glycosaminoglycan binding
growth factor receptor binding
MHC protein binding
olfactory receptor activity
hijacked molecular function
fibronectin binding
proteoglycan binding
cytokine receptor activity
cytokine receptor binding
histone acetyltransferase binding
collagen binding
antigen binding
coreceptor activity
extracellular matrix structural constituent
cytokine binding
0 0.5 1 1.5 2 2.5
P < 0.001 P < 0.01 P < 0.05 normalized_Enrichment_Score

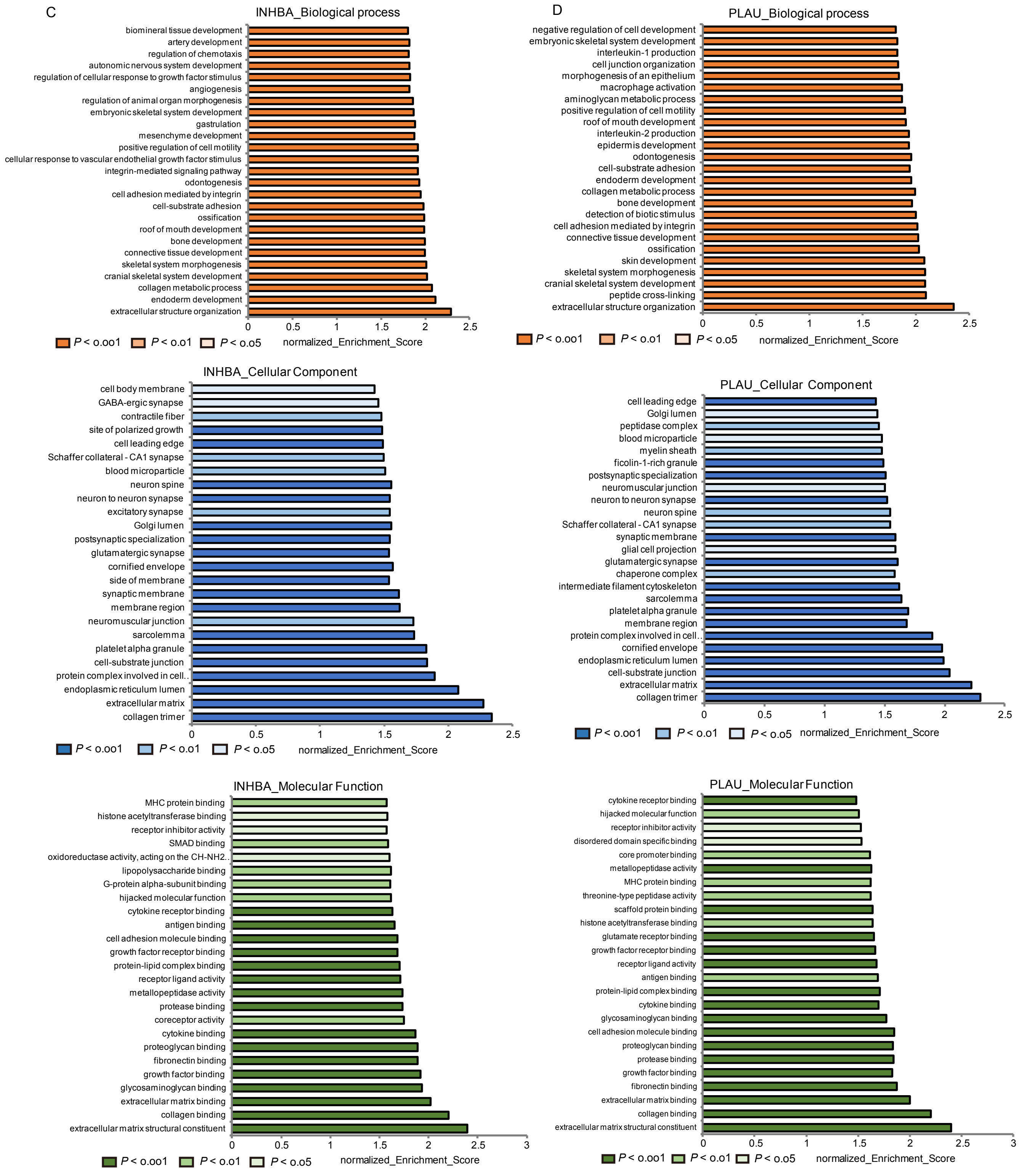

Figure s4. GO analyses of *CXCL10, TNFAIP3, INHBA,* and *PLAU*. (A-D) GO (biological process, cellular component, molecular function) terms related to co-expressed genes of *CXCL10* (A), *TNFAIP3* (B), *INHBA* (C), and *PLAU* (D) in the TCGA ESCC dataset.
