# Supplementary material for: Identifying the p65-Dependent Effect of Sulforaphene on Esophageal Squamous Cell Carcinoma Progression via Bioinformatics Analysis
Source: Int J Mol Sci. 2020 Dec 23;22(1):60. doi: 10.3390/ijms22010060 (PMC7793474; doi:10.3390/ijms22010060)
Supplement: Supplementary file 1 [file ijms-22-00060-s001.zip › supplementary figure_s5.PDF.pdf]

A

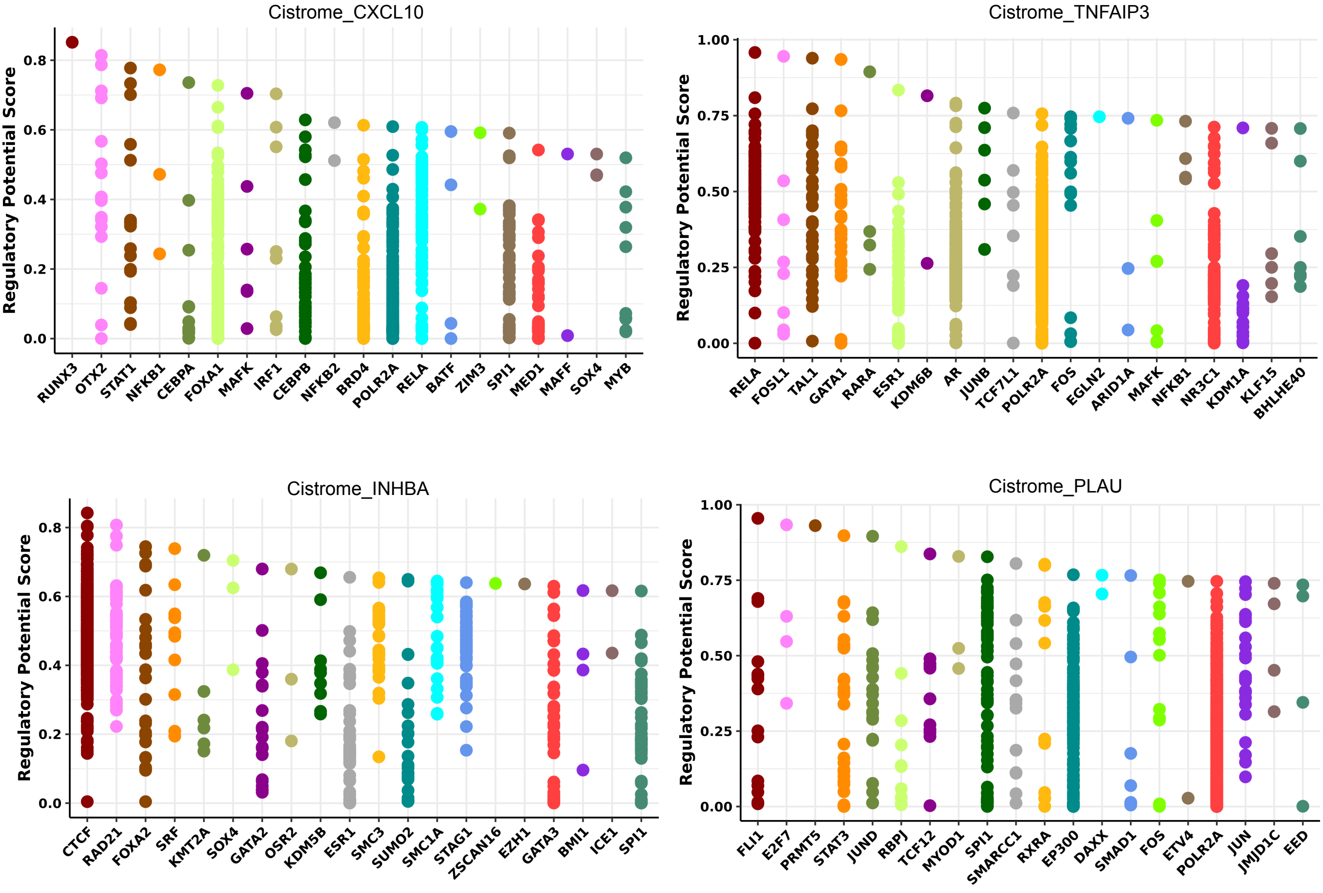
Cistrome_CXCL10
Regulatory Potential Score
0.0
0.2
0.4
0.6
0.8
RUNX3
OTX2
STAT1
NFKB1
CEBPA
FOXA1
MAFK
IRF1
CEBPB
NFKB2
BRD4
POLR2A
RELA
BATF
ZIM3
SPI1
MED1
MAFF
SOX4
MYB
Cistrome_TNFAIP3
Regulatory Potential Score
0.00
0.25
0.50
0.75
1.00
RELA
FOSL1
TAL1
GATA1
RARA
ESR1
KDM6B
AR
JUNB
TCF7L1
POLR2A
FOS
EGLN2
ARID1A
MAFK
NFKB1
NR3C1
KDM1A
KLF15
BHLHE40
Cistrome_INHBA
Regulatory Potential Score
0.0
0.2
0.4
0.6
0.8
CTCF
RAD21
FOXA2
SRF
KMT2A
SOX4
GATA2
OSR2
KDM5B
ESR1
SMC3
SUMO2
SMC1A
STAG1
ZSCAN16
EZH1
GATA3
BMI1
ICE1
SPI1
Cistrome_PLAU
Regulatory Potential Score
0.00
0.25
0.50
0.75
1.00
FLI1
E2F7
PRMT5
STAT3
JUND
RBPJ
TCF12
MYOD1
SPI1
SMARCC1
RXRA
EP300
DAXX
SMAD1
FOS
ETV4
POLR2A
JUN
JMJD1C
EED

B Cistrome_UCSC_hg38_CXCL10

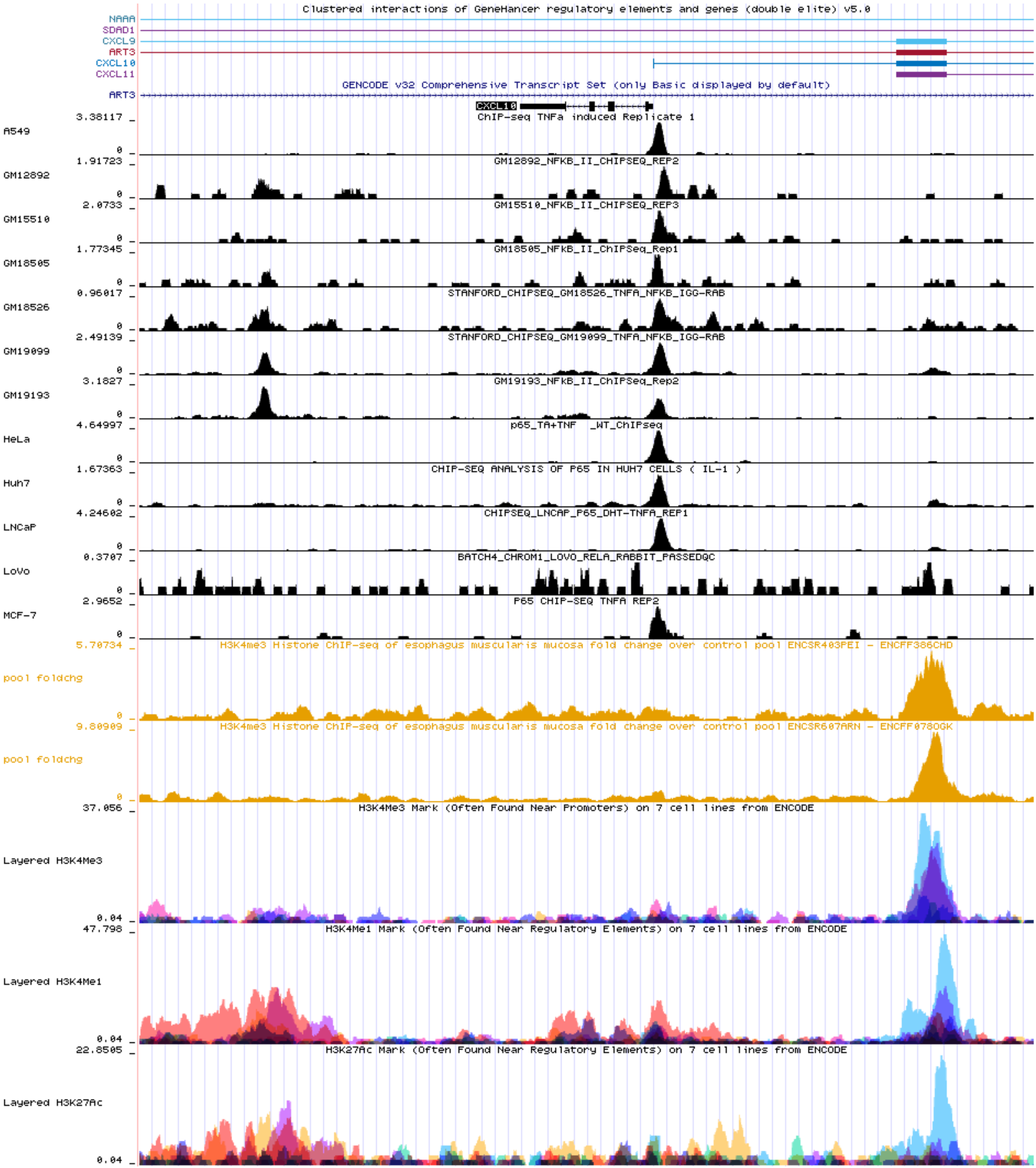

C Cistrome_UCSC_hg38_TNFAIP3

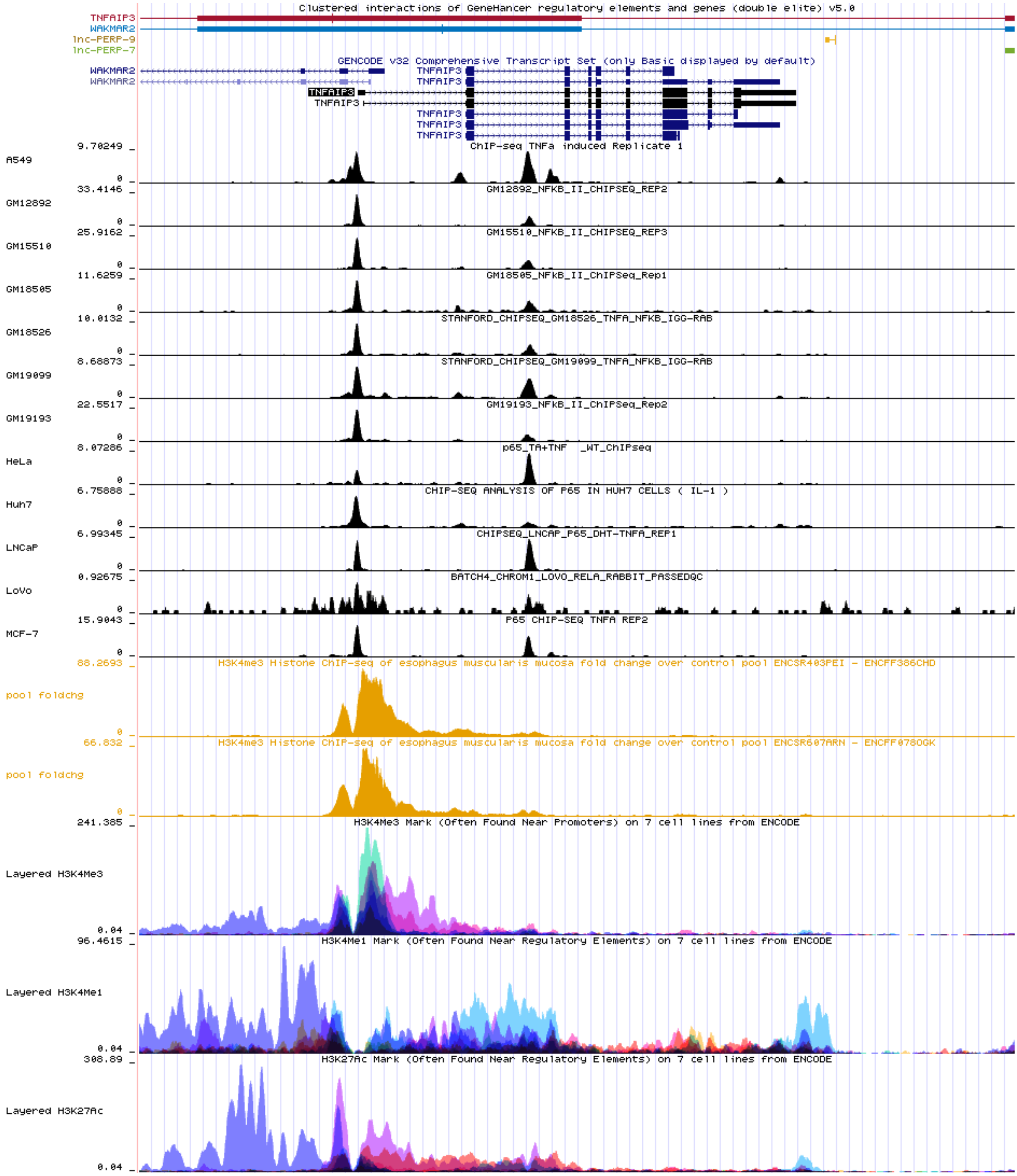

D

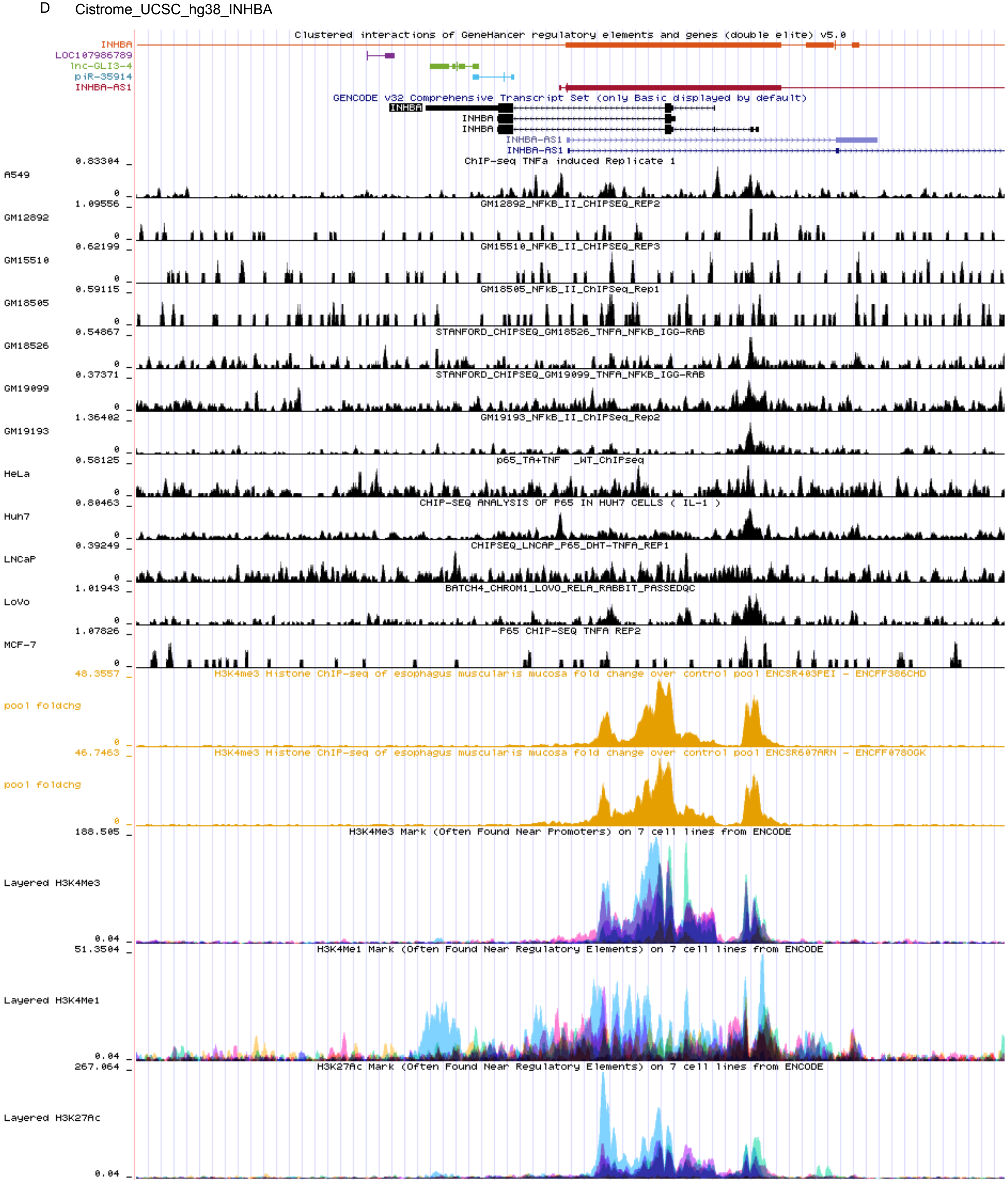
Cistrome_UCSC_hg38_INHBA
Clustered interactions of GeneHancer regulatory elements and genes (double elite) v5.0
INHBA
LOC107986789
lnc-GLI3-4
piR-35914
INHBA-AS1
GENCODE v32 Comprehensive Transcript Set (only Basic displayed by default)
INHBA
INHBA
INHBA
INHBA-AS1
INHBA-AS1
A549
ChIP-seq TNFa induced Replicate 1
GM12892
GM12892_NFKB_II_CHIPSEQ_REP2
GM15510
GM15510_NFKB_II_CHIPSEQ_REP3
GM18505
GM18505_NFkB_II_ChIPSeq_Rep1
GM18526
STANFORD_CHIPSEQ_GM18526_TNFA_NFKB_IGG-RAB
GM19099
STANFORD_CHIPSEQ_GM19099_TNFA_NFKB_IGG-RAB
GM19193
GM19193_NFKB_II_ChIPSeq_Rep2
HeLa
p65_TA+TNF _WT_ChIPseq
Huh7
CHIP-SEQ ANALYSIS OF P65 IN HUH7 CELLS ( IL-1 )
LNCaP
CHIPSEQ_LNCAP_P65_DHT-TNFA_REP1
LoVo
BATCH4_CHROM1_LOVO_RELA_RABBIT_PASSEDQC
MCF-7
P65 CHIP-SEQ TNFA REP2
pool foldchg
H3K4me3 Histone ChIP-seq of esophagus muscularis mucosa fold change over control pool ENCSR403PEI - ENCFF386CHD
pool foldchg
H3K4me3 Histone ChIP-seq of esophagus muscularis mucosa fold change over control pool ENCSR607ARN - ENCFF078OGK
Layered H3K4Me3
H3K4Me3 Mark (Often Found Near Promoters) on 7 cell lines from ENCODE
Layered H3K4Me1
H3K4Me1 Mark (Often Found Near Regulatory Elements) on 7 cell lines from ENCODE
Layered H3K27Ac
H3K27Ac Mark (Often Found Near Regulatory Elements) on 7 cell lines from ENCODE

E Cistrome_UCSC_hg38_PLAU

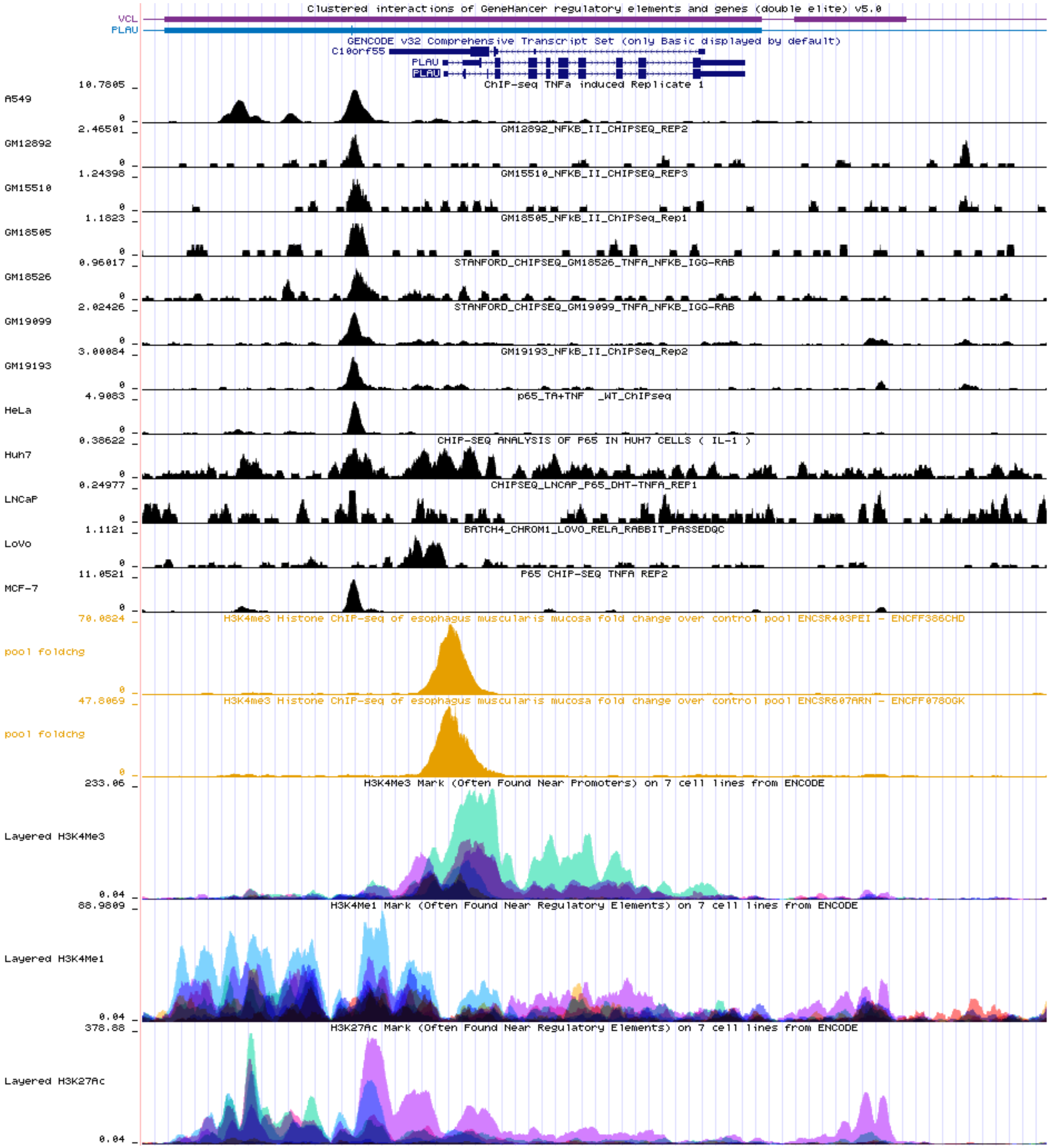


**Figure s5. NFκB p65 regulates the transcription of *CXCL10*, *TNFAIP3*, *INHBA* and *PLAU*.** (A) Based on ChIP-seq, DNase-seq, and ATAC-seq data collected in the Cistrome database, the top 20 transcription factors of *CXCL10, TNFAIP3. INHBA* and *PLAU* are shown. A parameter of 100kb regulatory potential decay rate was selected. Each dot represents a ChIP-seq sample. The transcription factors were ranked by the maximum regulatory potential score over all ChIP-seq samples. (B-E) ChIP-seq data of p65 with multiple biological sources from the Cistrome database was aligned to the GRCh38/hg38 reference assembly using UCSC Browser. The regulation of p65 on *CXCL10* (B), *TNFAIP3* (C), *INHBA* (D), and *PLAU* (E), and histone ChIP-seq data of 6 cell lines and esophagus muscularis mucosa samples are shown.
