# Supplementary material for: Identifying the p65-Dependent Effect of Sulforaphene on Esophageal Squamous Cell Carcinoma Progression via Bioinformatics Analysis
Source: Int J Mol Sci. 2020 Dec 23;22(1):60. doi: 10.3390/ijms22010060 (PMC7793474; doi:10.3390/ijms22010060)
Supplement: Supplementary file 1 [file ijms-22-00060-s001.zip › supplementary figure_s6.PDF.pdf]

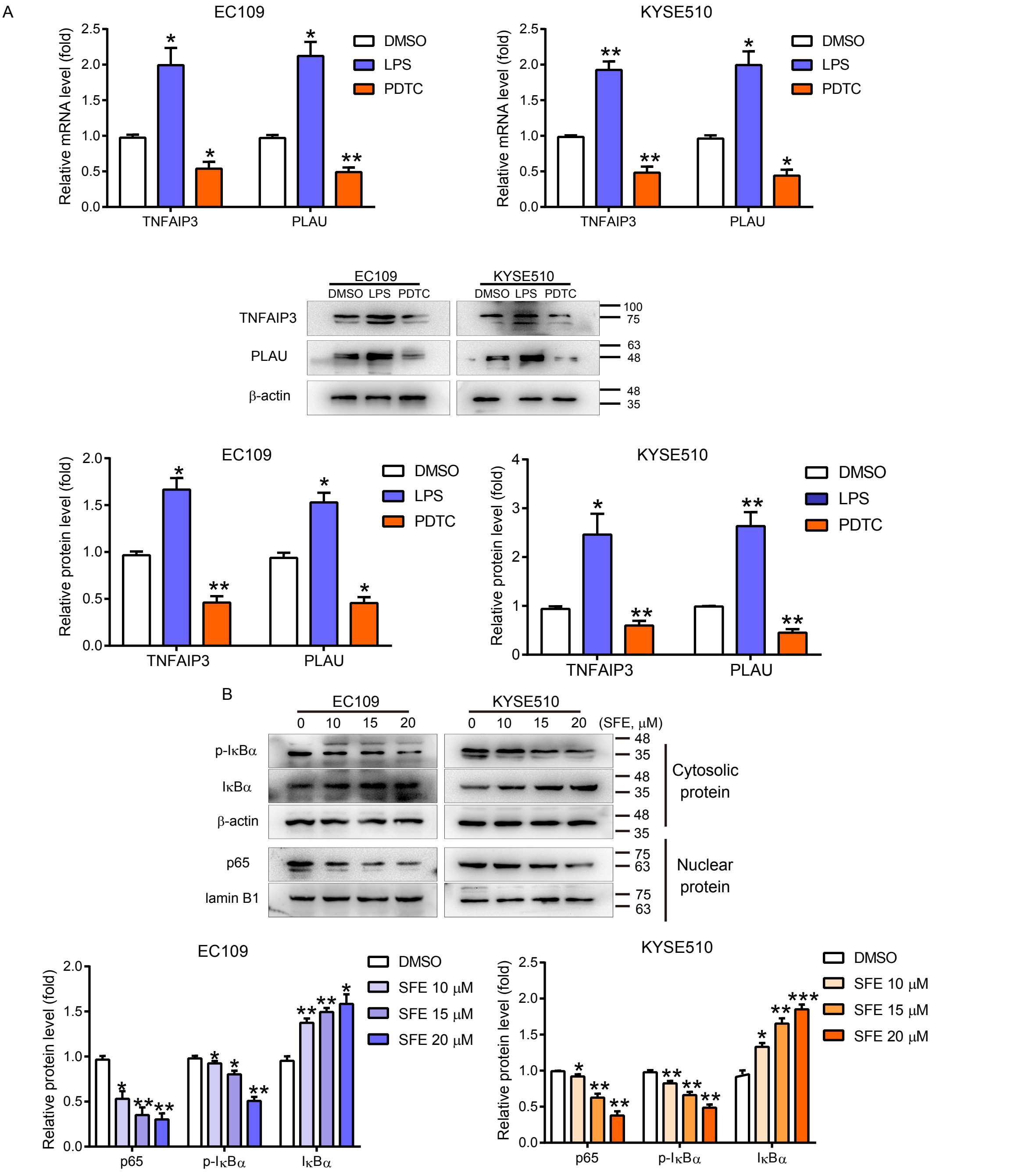

Figure s6. SFE inactivates the NFκB pathway to reduce *TNFAIP3* and *PLAU* expression.
(A) The NFκB pathway-related protein levels in SFE-treated ESCC cells. (B) The mRNA and protein levels of *TNFAIP3* and *PLAU* in ESCC cells with LPS or PDTC treatment. The statistical significance was assessed by Student's *t*-test. * $P < 0.05$, ** $P < 0.01$, and *** $P < 0.005$.
