# Supplementary material for: Identifying the p65-Dependent Effect of Sulforaphene on Esophageal Squamous Cell Carcinoma Progression via Bioinformatics Analysis
Source: Int J Mol Sci. 2020 Dec 23;22(1):60. doi: 10.3390/ijms22010060 (PMC7793474; doi:10.3390/ijms22010060)
Supplement: Supplementary file 1 [file ijms-22-00060-s001.zip › supplementary figure_s7.PDF.pdf]

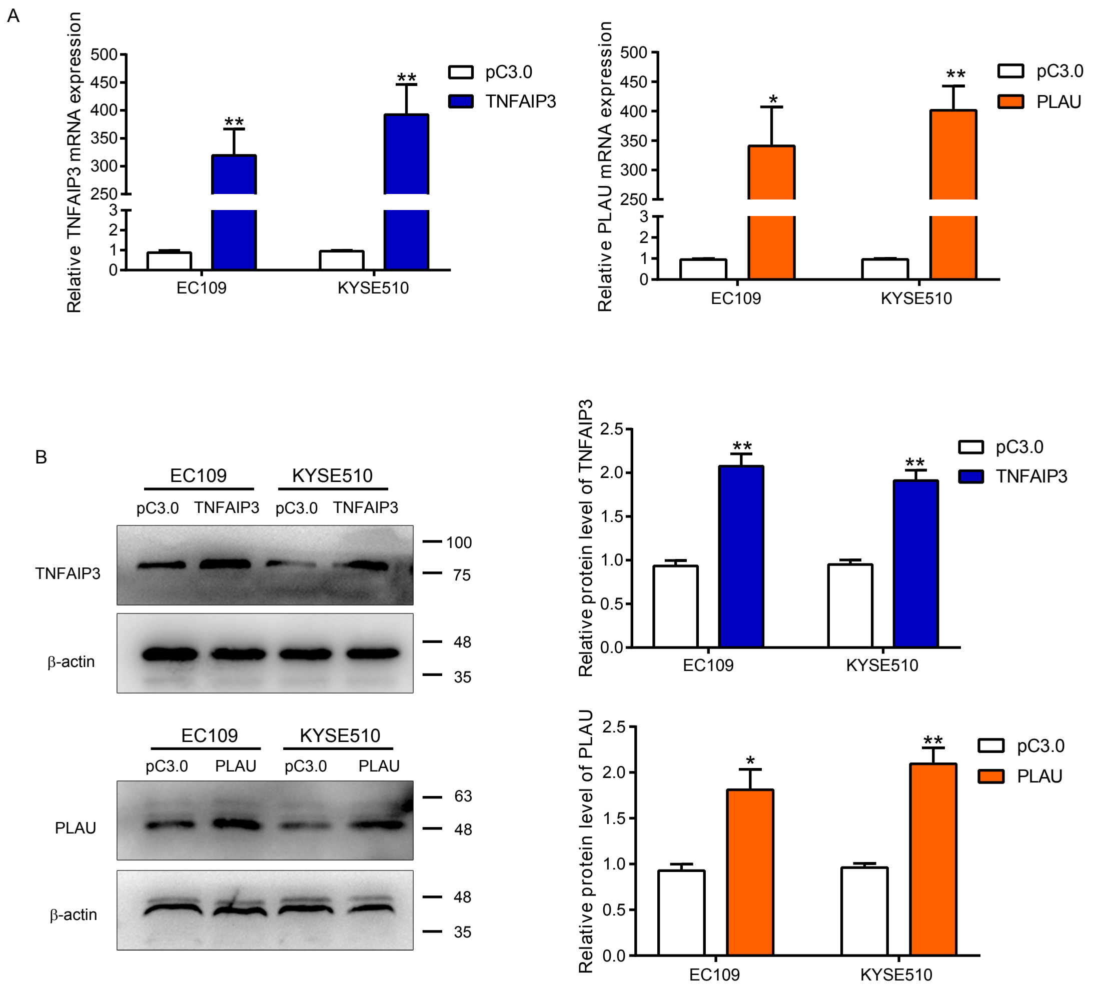

Figure s7. The effect of pC3.0-TNFAIP3 and pC3.0-PLAU transfected on *TNFAIP3* and *PLAU* expression. (A-B) The mRNA (A) and protein (B) levels of *TNFAIP3* and *PLAU* in ESCC cells transfected with pC3.0-TNFAIP3 (TNFAIP3) and pC3.0-PLAU (PLAU). The statistical significance was assessed by Student's *t*-test. * $P < 0.05$ and ** $P < 0.01$.
