# Supplementary material for: Identifying the p65-Dependent Effect of Sulforaphene on Esophageal Squamous Cell Carcinoma Progression via Bioinformatics Analysis
Source: Int J Mol Sci. 2020 Dec 23;22(1):60. doi: 10.3390/ijms22010060 (PMC7793474; doi:10.3390/ijms22010060)
Supplement: Supplementary file 1 [file ijms-22-00060-s001.zip › Table S1.docx]

**Table S1. Primer sequence for qPCR and ChIP-PCR**

qPCR primers:

|  | Forward Sequence (5'→3') | Reverse Sequence (5'→3') |
| --- | --- | --- |
| CXCL10 | GTGGCATTCAAGGAGTACCTC | TGATGGCCTTCGATTCTGGATT |
| TNFAIP3 | GCCCAGGAATGCTACAGATAC | AGTGGAACAGCTCGGATTTC |
| INHBA | CAAGAGCCAGGAAGAAAC | TGGTCCACTCTCAATCAC |
| PLAU | TGACCCACAGTGGAAAACAG | TTGTCCTTCAGGGCACATC |
| PLAUR | TGTAAGACCAACGGGGATTGC | AGCCAGTCCGATAGCTCAGG |
| CXCL1 | CCCAAACCGAAGTCATAGCCA | GATGCAGGATTGAGGCAAGC |
| CXCL11 | GACGCTGTCTTTGCATAGGC | GGATTTAGGCATCGTTGTCCTTT |
| CCL20 | TGCTGTACCAAGAGTTTGCTC | CGCACACAGACAACTTTTTCTTT |
| β-actin | GGCGGCACCACCATGTACCCT | AGGGGCCGGACTCGTCATACT |

ChIP-PCR primers:

|  | Forward Sequence (5'→3') | Reverse Sequence (5'→3') |
| --- | --- | --- |
| TNFAIP3 (-89/-410) | CCCGGAGAAACTCCTAGGTC | GCCGCTTTTTCTGTCAATTC |
| TNFAIP3 (+5936/+6047) | GCTGTTGCTCAATTGCTAGTC | CTTCTTGTGCTTACTTTCAGTTCTT |
| PLAU (-1955/-1729) | CTCTCAGCAATCAGCATGAC | TCCTCTAGAAGACTGTGGTCAG |
