# Supplementary material for: Identifying the p65-Dependent Effect of Sulforaphene on Esophageal Squamous Cell Carcinoma Progression via Bioinformatics Analysis
Source: Int J Mol Sci. 2020 Dec 23;22(1):60. doi: 10.3390/ijms22010060 (PMC7793474; doi:10.3390/ijms22010060)
Supplement: Supplementary file 1 [file supplementary figure_s2.PDF.pdf › supplementary figure_s2.PDF.pdf]

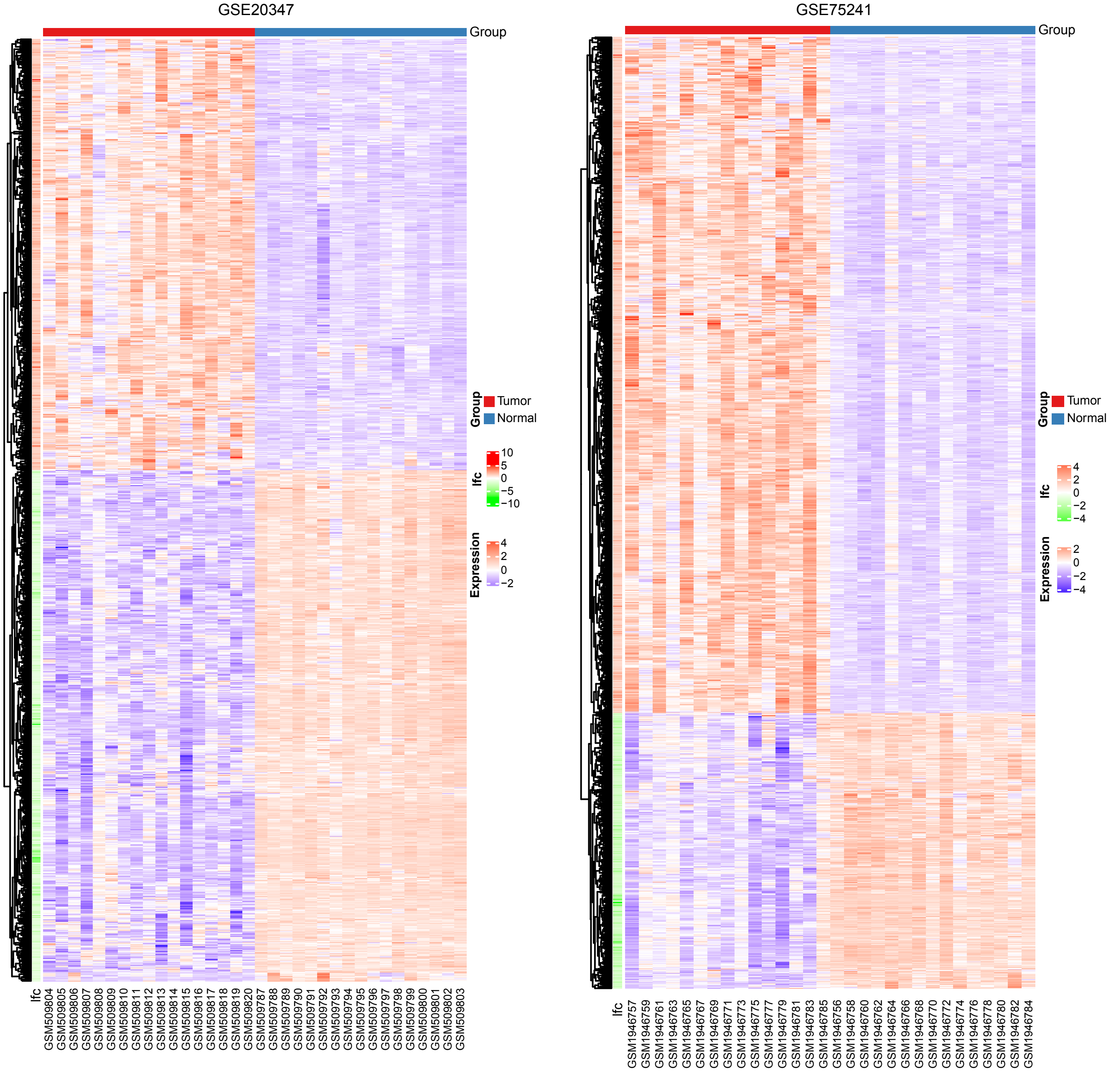

Figure s2. Analysis of GSE20347 and GSE75241 dataset. Heat maps show the differential expression genes in GSE20347 and GSE75241. lfc, log 2(fold change)
