# Supplementary material for: Identifying the p65-Dependent Effect of Sulforaphene on Esophageal Squamous Cell Carcinoma Progression via Bioinformatics Analysis
Source: Int J Mol Sci. 2020 Dec 23;22(1):60. doi: 10.3390/ijms22010060 (PMC7793474; doi:10.3390/ijms22010060)
Supplement: Supplementary file 1 [file ijms-22-00060-s001.zip › Table S2.doc]

**Table S2. Primary antibodies used in this study.**

| Name | Catalog number | Manufacture | |
| --- | --- | --- | --- |
| TNFAIP3 | 4625 | Cell Signaling Technology | Danvers, MA, USA |
| PLAU | ab169754 | Abcam | Cambridge, MA, USA |
| p-IκBα(Ser32) | 2859 | Cell Signaling Technology | Danvers, MA, USA |
| IκBα | 9242 | Cell Signaling Technology | Danvers, MA, USA |
| β-actin | 66009-1-Ig | Proteintech | Wuhan, China |
| p65 | 8242 | Cell Signaling Technology | Danvers, MA, USA |
| lamin B1 | 12987-1-AP | Proteintech | Wuhan, China |
